# Expanding the Scope of the Cleavable *N*-(Methoxy)oxazolidine Linker for the Synthesis of Oligonucleotide Conjugates

**DOI:** 10.3390/molecules26020490

**Published:** 2021-01-18

**Authors:** Aapo Aho, Antti Äärelä, Heidi Korhonen, Pasi Virta

**Affiliations:** Department of Chemistry, University of Turku, 20014 Turku, Finland; aamaah@utu.fi (A.A.); ankaaar@utu.fi (A.Ä.); hejoli@utu.fi (H.K.)

**Keywords:** oligonucleotide conjugates, cleavable linker, *N*-(methoxy)oxazolidine

## Abstract

Oligonucleotides modified by a 2′-deoxy-2′-(*N*-methoxyamino) ribonucleotide react readily with aldehydes in slightly acidic conditions to yield the corresponding *N*-(methoxy)oxazolidine-linked oligonucleotide-conjugates. The reaction is reversible and dynamic in slightly acidic conditions, while the products are virtually stable above pH 7, where the reaction is in a ‘‘switched off-state’’. Small molecular examinations have demonstrated that aldehyde constituents affect the cleavage rate of the *N*-(methoxy)oxazolidine-linkage. This can be utilized to adjust the stability of this pH-responsive cleavable linker for drug delivery applications. In the present study, Fmoc-β-Ala-H was immobilized to a serine-modified ChemMatrix resin and used for the automated assembly of two peptidealdehydes and one aldehyde-modified peptide nucleic acid (PNA). In addition, a triantennary *N*-acetyl-d-galactosamine-cluster with a β-Ala-H unit has been synthesized. These aldehydes were conjugated via *N-*(methoxy)oxazolidine-linkage to therapeutically relevant oligonucleotide phosphorothioates and one DNA-aptamer in 19–47% isolated yields. The cleavage rates of the conjugates were studied in slightly acidic conditions. In addition to the diverse set of conjugates synthesized, these experiments and a comparison to published data demonstrate that the simple conversion of Gly-H to β-Ala-H residue resulted in a faster cleavage of the *N*-(methoxy)oxazolidine-linker at pH 5, being comparable (T_0.5_ ca 7 h) to hydrazone-based structures.

## 1. Introduction

Oligonucleotide (ON) therapeutics, such as antisense oligonucleotides (ASO) and small interfering RNAs (siRNAs), can be applied for the modulation of gene expression in a wide range of disorders [1,2,3,4,5,6,7,8,9]. Despite the great potential of ONs as drugs, they suffer from poor pharmacokinetic properties [10]. Backbone modifications such as phosphorothioate and 2′-*O*-substitutions improve the stability and increase the plasma circulation time of Ons [10,11], but cell/tissue-specific extrahepatic delivery has remained a challenge [8,12]. For targeted delivery, antibodies [13,14,15], aptamers [16,17], nanoparticles [18], extracellular vesicles [19], carbohydrates [20,21,22], cholesterol [23,24] m and other small molecules [25,26,27] have been utilized. However, almost without exception these strategies lead to the endosomal entrapment of ONs [15,18,28]. Endosomal escape may be facilitated by other structural modifications or conjugate groups [29,30,31], which may make the overall synthesis complex. In the synthesis of these biomolecular hybrids, in which even the bis-conjugation of ONs is needed, orthogonal ligation chemistries play a central role. It is beneficial if the conjugation itself creates a linker that is cleavable [32,33,34]. The linker should also provide efficient conjugation, be stable in physiological conditions, and release the therapeutic ON cargo in appropriate intracellular compartments. Examples of such linkers are hydrazones [35,36], which are cleaved in slightly acidic conditions perceived to that in endosomes and lysosomes, and disulfides [14,16], which are cleaved in a mildly reducible environment in cytosol. Hence, the former linker chemistry may be suitable for the conjugation of cell/tissue targeting vehicles, whereas the latter may be suitable for the conjugation of endosomal escaping moieties. Expanding the chemistry of reversible linkers is important to find efficient orthogonal conjugation strategies and the targeted release of ON therapeutics in biological mediums.

**Figure 1:**
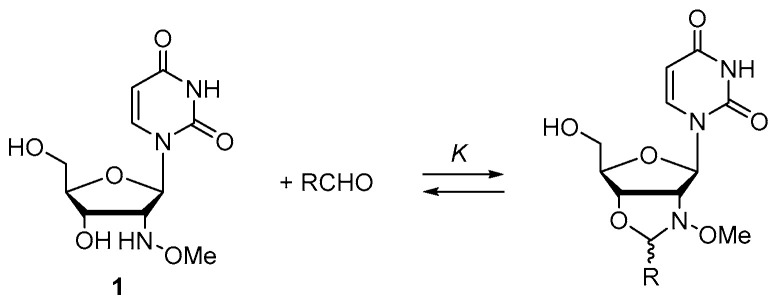
Formation of *N-*(methoxy)oxazolidines between 2′-deoxy-2′-(*N*-methoxyamino) uridine and small molecule aldehydes (R = cf. Table 2).

**Scheme 1:**
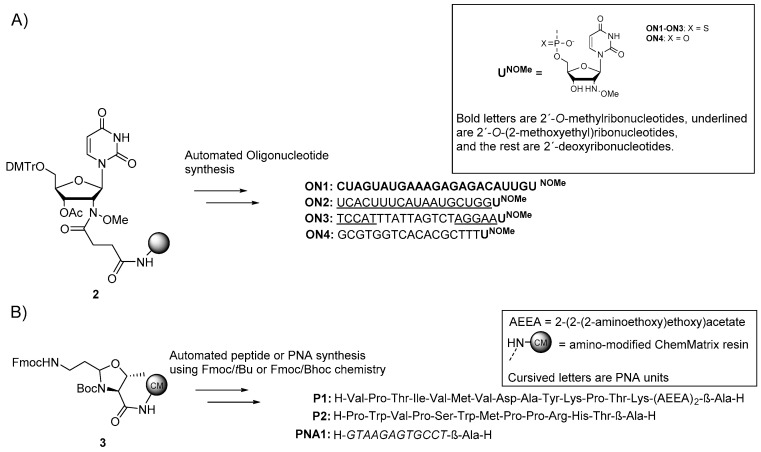
(**A**) Automated synthesis of **U^NOMe^** -elongated oligonucleotides (**ON1**, **ON2**, **ON3**, and **ON4**) using U^NOMe^ solid support. (**B**) Automated synthesis of β-Ala-H-modified peptides (**P1** and **P2**) and PNA (**PNA1**) using Fmoc-β-Ala-H bound on an amino-modified ChemMatrix resin via *N*-Boc-oxazolidine.

Recently, the reversible formation of *N*-(methoxy)oxazolidine (Figure 1) was employed in conjugation between 2′-deoxy-2′-(*N*-methoxyamino)uridine (**U^NOMe^**, Scheme 1a)-modified ONs and Gly-H-modified peptide aldehydes [37]. The **U^NOMe^**-ONs and peptide aldehydes were both synthesized by automated assembly using appropriately modified solid supports. After cleavage, deprotection, and purification, the **U^NOMe^**-ONs and the peptide aldehydes were mixed in slightly acidic conditions to yield conjugates in reasonable yields. The conjugates were stable during RP HPLC purification and lyophilization, but showed an acid-dependent hydrolytic cleavage. ONs were released from Gly-H-modified peptide aldehydes with a half-life (t_0.5_) of 5.8, 42, and 220 h at pH 4, 5, and 6, respectively (37 °C) (cf. Table 1 entries 1–3), and only 11% was released after two weeks of incubation at pH 7 (37 °C). It was additionally shown by small molecular models that the rate of the *N*-(methoxy)oxazolidine hydrolysis could be adjusted using structurally different aldehydes.

In this study, the scope of the *N*-(methoxy)oxazolidine ligation was expanded by the synthesis of a more diverse set of conjugates. **U^NOMe^**-extended ONs, consisting of three therapeutically relevant ONs, ISE-AR-V7 [38], Nusinersen [39], IONIS-DGAT2_RX_ [40,41], and one DNA-aptamer, TfRA3 [42], were synthesized and conjugated to four different β-Ala-H-containing biomolecules: SpyTag [43], D-retro inverso THR [44,45,46], an antisense PNA [47], and a trivalent *N*-acetyl galactosamine (GalNAc) cluster (cf. further description of these biomolecules below). The ligation products could be obtained in 19–47% isolated yields. The hydrolysis rate of the conjugates (i.e., reverse ligation) was studied at pH 4, 5, 6, and 7.4. The *N*-(methoxy)oxazolidine linker, bound to the β-Ala-H residue, clearly cleaved faster in acidic conditions (pH 4–6) than the previously studied Gly-H-based conjugates [37]. The cleavage was comparable to most hydrazone linkers, and the conjugates maintained their hydrolytic stability in physiological conditions (pH 7.4).

## 2. Results

### 2.1. Small Molecular Model Study 

Prior to real conjugation experiments (described below), the *N*-(methoxy)oxazolidine formation with a β-Ala-H residue was studied using small-molecule models. 2′-deoxy-2′-(*N*-methoxyamino)uridine (**1,** 5 mM) and *N*-Bz-β-Ala-H (5 mM) were mixed in buffered aqueous solution (pH 4) at room temperature and the progress of the reaction was followed by RP HPLC. As expected, two *N*-(methoxy)oxazolidine ligation products (R/S isomers) were formed (cf. RP HPLC profile of the reaction and characterization of the products in Supplementary Materials). The reaction stalled at equilibrium (*K* = 2.82 ± 0.43 × 10^3^ L mol^−1^), yielding a 75% conversion of **1** to the ligation products. The hydrolysis rate of the obtained *N*-(methoxy)oxazolidine was determined at pH 4, 5, and 6 by following the degradation of the major ligation product. As expected, the hydrolysis rate was pH-dependent, with half-lives of 5.3, 29, and 310 h at pH 4, 5, and 6, respectively, being ca. three-fold faster than the hydrolysis of *N*-Bz-Gly-H ligation product (Figure 1 and Table 2). Despite the modest rate enhancement of the hydrolysis, this model reaction was well-behaving and promising, considering the conjugation of ONs with β-Ala-H-containing biomolecules.

### 2.2. Synthesis of 2′-deoxy′-2′-(N-methoxyamino)uridine-Modified Oligonucleotides

Four oligonucleotides elongated by **U^NOMe^** (AON-ISE-AR-V7-**U^NOMe^** (**ON1**), Nusinersen-**U^NOMe^** (**ON2**), IONIS-DGAT2_RX_-**U^NOMe^** (**ON3**), and TfRA3-**U^NOMe^** (**ON4**) (Scheme 1a) were next synthesized using previously prepared solid support **2** [37], commercial phosphoramidite building blocks, and automated chain assembly. AON-ISE-AR-V7 suppresses prostate tumor cell survival by the inhibition of androgen receptor variant 7 mRNA synthesis [38], Nusinersen is an approved drug used for treating spinal muscular athropy [39], and IONIS-DGAT2_RX_ ASO reduces DGAT2 enzyme production and is a potential treatment for nonalcoholic steatohepatitis [40,41]. TfRA3 is a transferring receptor-binding aptamer [41] that can act as a delivery vehicle to traverse the blood brain barrier (BBB) (i.e., the role of this ON in the cargo-delivery vehicle construct **C4** is inverse compared to that of conjugates **C1**–**C3**, cf below). After chain elongation, cleavage with concentrated aqueous ammonia followed by RP HPLC purification gave the desired oligonucleotides in 29–40% yields (cf. supporting information).

### 2.3. Synthesis of β-Ala-H-Modified Biomolecules

Two peptide aldehydes and one PNA aldehyde were synthesized by following a published protocol [48,49]. Fmoc-β-Ala-H was bound to an amino-modified ChemMatrix resin via *N*-(Boc)oxazolidine to obtain solid support **3**. On this support, SpyTag-(AEEA)_2_-β-Ala-H (**P1**), retro inverso THR-β-Ala-H (**P2**), and GluR3 antisense PNA-β-Ala-H (**PNA1**) were synthesized using automated Fmoc-chemistry (Scheme 1b). SpyTag peptide binds through irreversible isopeptide bond to a SpyCatcher protein domain [43]. This autocatalytic process has been utilized, e.g., for the preparation of antibody-ON-conjugates [50], but immunogenicity issues should be resolved prior to drug delivery applications. THR and its peptidase-resistant retro inverso version [44] binds to the transferrin receptor, and it has been applied to deliver RNA nanoparticles through the blood brain barrier [45,46]. GluR3 antisense PNA has been shown to reduce the glutamate excitotoxicity associated with amyotrophic lateral sclerosis (ALS) by reducing GluR3 protein expression [47]. After chain elongation, the peptides/PNA were cleaved from the resin using a TFA cocktail (cf. supporting information for more details); precipitated in cold ether; and dissolved in aq. 0.01% TFA to yield **P1**, **P2**, and **PNA1**, which were purified by RP HPLC (cf. supporting information).

**Scheme 2:**
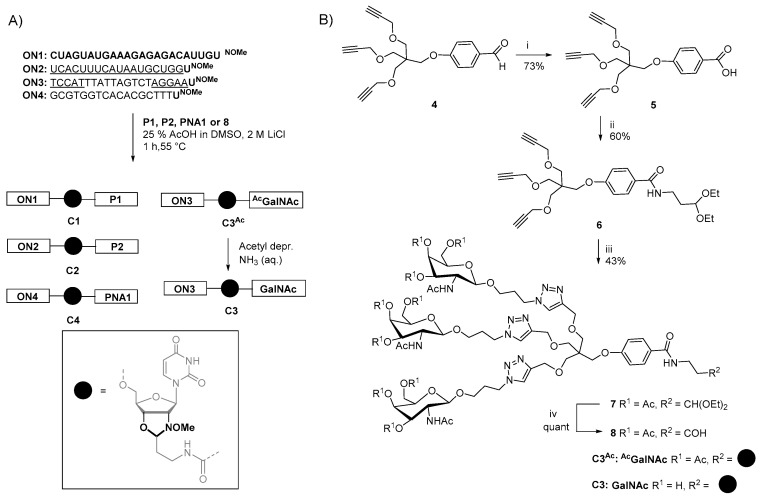
(**A**) Synthesis of the conjugates **C1**, **C2**, **C3,** and **C4**. (**B**) Synthesis of the trivalent GalNAc conjugate group **8**. Reaction conditions: (i) 3 equiv. Jones reagent, MeCN, 1 h at rt; (ii) 1.1 equiv. 3-amino-1,1-diethoxypropane, 2.5 equiv. *N,N*-diisopropyl ethylamine (DIPEA) 1.1 equiv BOP, DMF, on at rt; (iii) 5 equiv. (3-azidopropyl) 2-acetamido-3,4,6-tri-*O*-acetyl-2-deoxy-β-ᴅ-galactopyranoside, catalytic Cu(I)I, DMF/dioxane/H_2_O (3:3:1, *v*/*v*/*v*), overnight at 40 °C; (iv) aq. 0.01% TFA, 20 min at 55 °C.

One β-Ala-H-containing trivalent GalNAc cluster, with a good potential for liver targeting via asialoglycoprotein receptor [51], was additionally synthesized starting from branching unit **4** [52] consisting of three alkynyl and one aromatic aldehyde group (Scheme 2b). First, the aldehyde moiety was oxidized by Jones’ condition using Cr_3_O. The resulting carboxylic acid (**5**) was coupled to the diethoxy acetal of β-Ala-H using BOP/DIPEA activation to yield an amide (**6**). Then, the alkynyl groups of the core were coupled with (3-azidopropyl)-2-acetamido-3,4,6-tri-*O*-acetyl-2-deoxy-β-d-galactopyranoside using Cu(I) catalyzed 1,3-dipolar cycloaddition (i.e., click reaction). Finally, the acetal protection of **7** was removed by aq 0.01% TFA to expose the β-Ala-H functionality. The final product **8** was prepared in an 18% yield after four steps. It may be worth mentioning that in our previous study aryl aldehydes (cf. **4**) reacted only barely with **1**, and the β-Ala-H-extension (cf. **8**) is crucial to gain an efficient conjugation.

### 2.4. Synthesis of Oligonucleotide Conjugates C1-C4 Using N-(methoxy)oxazolidine Ligation

**Figure 2:**
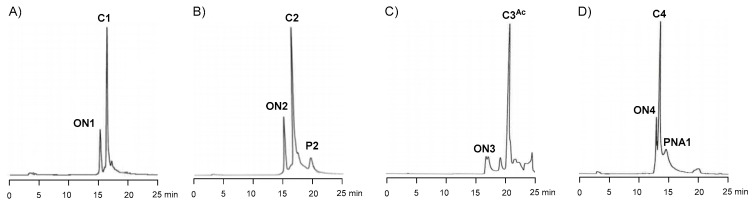
RP HPLC (C18, 250 × 4.6 mm, 5 µm) profiles of *N*-methoxyoxazolidine conjugation crude product mixtures. A linear gradient of MeCN over aq. triethylammonium acetate (TEAA) buffer (pH 7) and a detection wavelength of 260 nm were employed. (**A**) Conjugate **C1,** 0–70% MeCN in 25 min; (**B**) Conjugate **C2**, 0–70% MeCN in 25 min; (**C**) **C3^Ac^**, 0–60% MeCN in 25 min; (**D**) **C4**, 0–70% MeCN in 25 min.

The rationale of the synthesized conjugates **C1**-**C4** below is based on the therapeutic relevance of the ASOs (cf. above) and the reported delivery potential of the corresponding conjugate groups to target tissues: **P2** and **ON4** could potentially enhance the CNS targeting of **ON2** and **PNA1** (i.e., **C2** and **C4**) and increase their potential as intravenously administrated drugs. However, nanoparticle-based delivery systems may be additionally needed in this approach. The GalNac cluster **8** could increase the liver targeting of **ON3** (**C3**). **P1** can readily be extended to an antibody construct specific to prostate membrane antigen (PMSA) and, in this way, improve the targeting of **ON1** (**C1**). The **U^NOMe^** oligonucleotides **ON1**, **ON2**, **ON3**, and **ON4** were mixed with an excess of the corresponding β-Ala-H conjugate groups **P1**, **P2**, **PNA1,** and **8** (Scheme 2a) and incubated in AcOH/DMSO (1:3, *v*/*v*), 2 M LiCl at 55 °C (reaction specific parameters in Table 3). After 1 h of incubation, the reaction mixtures were neutralized using dilute aq. NaOH and subjected as such to RP HPLC. As seen in the RP HPLC profiles of the crude product mixtures (Figure 2), a good conversion of the products was obtained with a moderate excess of the aldehyde constituents (**P1**, **P2**, **PNA1,** and **8,** 2–8 equiv.). The product fractions were lyophilized to give the conjugates **C1**, **C2**, **C3^Ac^**, and **C4** in 19–47% yields (Table 3). Conjugate **C3^Ac^** was deacetylated by soaking the conjugate in concentrated aq. ammonia (3 h at rt), and, without further purification, lyophilized to give **C3**.

### 2.5. Hydrolysis of the Ligations Products

**Figure 3:**
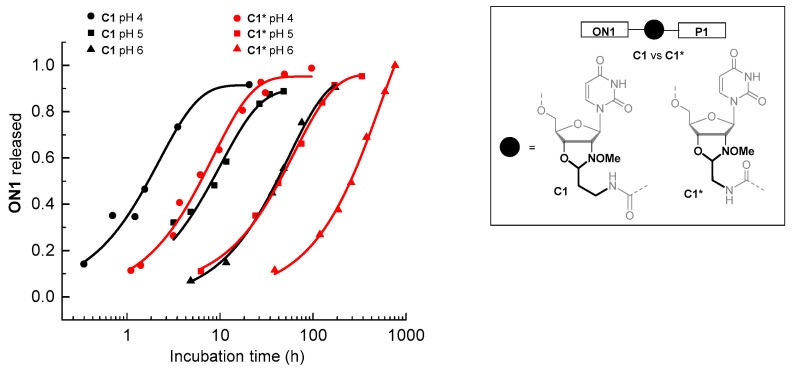
Comparison of the structures and hydrolysis rates of the analogous **C1** and C1*.

**Figure 4:**
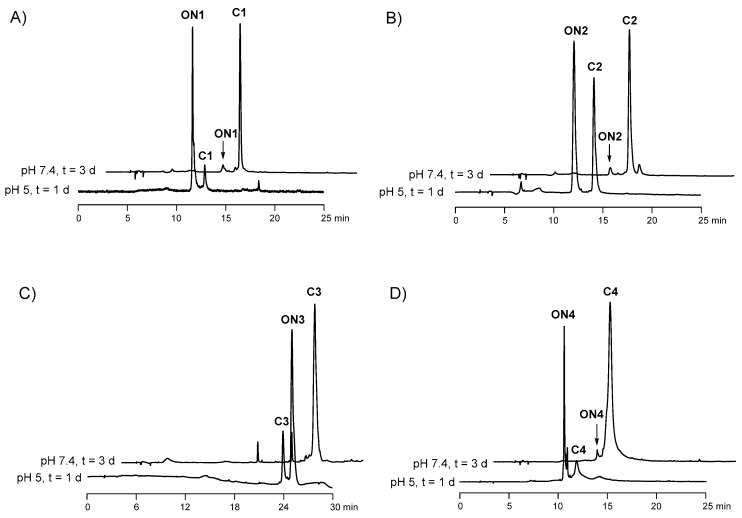
Selected RP HPLC (C18, 250 × 4.6 mm, 5 µm) profiles of the conjugate hydrolysis reactions. A detection wavelength of 260 nm was employed (**A**) **C1**, a linear gradient of 0–60% MeCN in 25 min over aq. TEAA buffer (pH 7); (**B**) **C2**, a linear gradient of 0–60% MeCN in 25 min over aq. TEAA buffer (pH 7); (**C**) **C3**, a linear gradient of 0–50% MeCN in 30 min over aq. hexylammonium acetate buffer (pH 7); (**D**) **C4**, 0–60% MeCN in 25 min over aq. TEAA buffer (pH 7).

The hydrolysis rates of the conjugates (**C1**–**C4**) were studied by incubating them (10 µM) in aq. buffers at 37 °C and monitoring the release of the ONs by RP HPLC (Figure 3 and Figure 4, Table 1). As predicted by the small-molecule models, **ON1** released from β-Ala-H-derived conjugate **C1** 5–6 times faster (at pH 5, t_0.5_ = 7.17 ± 0.77 h) than from Gly-H-derived conjugate C1* (at pH 5, t_0.5_ = 41.7 ± 2.3). Indeed, as illustrated in Figure 3, **C1** requires approximately one pH unit less acidic environment than C1* to reach the same rate of hydrolysis in the range of pH 4–6. Similarly, at pH 5 conjugates **C2**, **C3**, and **C4** were all hydrolyzed within t_0.5_ of 4.0–11.1 h (entries 7, 8, 9 in Table 1). Interestingly, there was variation in the hydrolysis rates and also in the equilibrium yields. The reaction is most likely affected by the macromolecular interactions (e.g., by electrostatic interactions between the ONs and positively charged peptides), and not only by the closest environment of the reaction center. All the conjugates were virtually stable at pH 7.4 after three days of incubation (Figure 4).

## 3. Discussion

The *N*-(methoxy)oxazolidine linker was found to be a reliable tool for conjugating **U^NOMe^**-extended ONs to a variety of β-Ala-H-containing biomolecules. The rate of hydrolysis of the *N*-(methoxy)oxazolidine conjugates (t_0.5_ = 4.4–10.2 at pH 5) was in the range of the currently used acid-labile linkers that are applied in antibody–drug conjugates. For example, a phenylketone-derived hydrazine linker used in gemtuzumab ozogamicin (Mylotarg) [53] and inotuzumab ozogamicin (Besponsa) [54] has been determined to hydrolyze 97% in 24 h at pH 4.5, which equals t_0.5_ = 4.74 h (according to first-order kinetics), and only 6% at pH 7.4 [53]. Obviously, the optimal release profiles of therapeutic ON conjugates may differ greatly from those of small-molecule drug conjugates. That said, the most central result here was that the release profile could be tuned by modifying the Gly-H aldehyde to a slightly less electron-deficient β-Ala-H without losing the convenience of the conjugate synthesis. It may be assumed that the release rate may be further accelerated using similar simple modifications. Furthermore, the *N*-(methoxy)oxazolidine conjugation was stable in concentrated ammonia. The option of removing the base-labile protecting groups post-conjugation may be useful in the synthesis of more complex conjugates or/and facilitating chromatographic issues.

## 4. Materials and Methods

### 4.1. 4-{3-(propynyloxy)-2,2-Bis [(propynyloxy)methyl]propoxy}benzoic Acid (**5**)

Compound **4** (78 mg, 0.22 mmol) was dissolved in MeCN (1.5 mL). A total of 1 equiv. of Jones reagent (CrO_3_ 22 mg, 0.22 mmol; 22 µL H_2_SO_4_; and H_2_O until the mixture is homogenous) was added to the mixture while stirring vigorously. The reaction was monitored by TLC (4% MeOH in DCM), and Jones reagent was added twice in 30 min intervals (1 + 1 equiv. of CrO_3_) until the oxidation was complete. The reaction was quenched by adding sat. aq. NaHCO_3_ (5 mL) and the product was extracted twice with DCM (2 × 5 mL). The organic phases were combined and washed twice with brine (5 mL). The organic phase was dried with Na_2_SO_4_, filtered, and evaporated to dryness to yield the product (**5**, 60 mg, 73%) as a transparent glassy substance. **^1^H NMR** δ_H_ (600 MHz, CDCl_3_): 8.07 (2H, d, *J* = 8.4 Hz), 6.98 (2H, d, *J* = 9.0 Hz), 4.16 (6H, d, *J* = 2.4 Hz), 4.08 (1H, s), 3.68 (6H, s), 2.42 (3H, t, *J* = 2.4 Hz). **^13^C NMR** δ_C_ (150 MHz): 171.7, 163.6, 132.2, 121.6, 114.4, 79.8, 74.3, 68.6, 66.9, 58.8, 44.8. **HRMS-ESI** (m/z) calc. for C_21_H_23_O_6_ [M+H^+^]^+^: 371.1495; found: 371.1492.

### 4.2. N-(3,3-diethoxypropyl)-4-{3-(propynyloxy)-2,2-bis [(propynyloxy)methyl]propoxy}benzamide (**6**)

BOP (54 mg, 0.11 mmol, predissolved in anhydrous DMF, 0.50 mL) was added to a mixture of compound **5** (37 mg, 0.10 mmol), 3-amino-1,1-diethoxypropane (18 µL, 0.11 mmol), and DIPEA (44 µL, 0.25 mmol) in DMF (0.50 mL). The reaction mixture was stirred overnight in room temperature and then quenched with sat. aq. NaHCO_3_ (5 mL). The product was extracted with EtOAc (2 × 5 mL). The organic phase was washed with sat. aq. NaHCO_3_ (3 mL) and brine (3 mL), dried with Na_2_SO_4_, filtered, and evaporated to dryness. The crude product was purified by silica gel chromatography (6% MeOH in DCM) to yield the product (**6**, 31 mg, 60%) as a white foam. **^1^H NMR** δ_H_ (600 MHz, CDCl_3_) 7.72 (2H, d, *J* = 8.4 Hz), 6.94 (2H, d, *J* = 9.0 Hz), 4.65 (1H, t, *J* = 5.4 Hz), 4.15 (6H, d, *J* = 2.4 Hz), 4.04 (1H, s), 3.74 (2H, m), 3.67 (6H, s), 3.58 (2H, m), 3.55 (2H, m), 2.41 (3H, t, *J* = 2.4 Hz), 1.96 (2H, m), 1.26 (6H, t, *J* = 7.2 Hz).**^13^C NMR** δ_C_ (150 MHz): 166.6, 161.6, 128.4, 127.1, 114.4, 103.2, 79.8, 74.3, 68.7, 66.8, 62.2, 58.8, 44.8, 35.9, 32.8, 15.4. **HRMS-ESI** (m/z) calc. for C_28_H_37_NNaO_7_ [M+Na^+^]^+^: 522.2468; found 522.2460.

### 4.3. Diethoxy Acetal-Protected Trivalent GalNAc Cluster (**7**)

(3-azidopropyl) 2-acetamido-3,4,6-tri-*O*-acetyl-2-deoxy-β-ᴅ-galactopyranoside (88 mg, 0.20 mmol) [55] and compound **6** (15 mg, 0.041 mmol) were dissolved in a mixture of DMF/dioxane/H_2_O (3:3:1, *v*/*v*/*v,* 0.60 mL). A crystal of CuI was added and the mixture was stirred overnight on oil bath (40 °C). The reaction was quenched by adding aq. EDTA (0.1 M, 4 mL). The product was extracted with DCM (12 mL). The organic phase was washed with aq. EDTA (0.1 M, 4 mL), H_2_O (4 mL), and brine (4 mL), then dried with Na_2_SO_4_, filtered, and evaporated to dryness. The crude product was purified by silica gel chromatography (6–10% MeOH in DCM) to yield the product (**7**, 32 mg, 43%) as a white foam. ^1^H-NMR δ_H_ (600 MHz, CDCl_3_): 7.65 (2H, d, *J* = 8.4 Hz), 7.57 (2H, s), 6.79 (2H, d, *J* = 9.0 Hz), 5.28 (3H, d, *J* = 3.0 Hz), 5.02 (3H, dd, *J* = 11.0 Hz & 3.0 Hz), 4.50 (6H, s), 4.48–4.36 (7H, m), 4.29 (3H, m), 4.11 (3H, dd, *J* = 9.9 Hz & 8.4 Hz), 4.07 (6H, m), 3.88 (2H, s), 3.84 (6H, m), 3.62 (4H, q, *J* = 7.2 Hz), 3.51 (6H, s), 3.44 (2H, t, *J* = 5.4 Hz), 3.28 (3H, m), 2.10 (12H, m), 1.98 (12H, m), 1.95 (9H, m), 1.90 (11H, m), 1.16 (6H, t, *J* = 7.2 Hz). ^13^C-NMR δ_C_ (150 MHz): 160.7, 170.6, 167.3, 161.5, 144.8, 128.6, 123.4, 123.4, 114.2, 103.9, 101.5, 70.5, 68.5, 66.6, 65.4, 64.4, 61.4, 57.9, 50.3, 46.7, 45.0, 35.7, 31.8, 30.1, 22.9, 20.5, 18.0. MS-ESI (*m*/*z*) calc. for C_77_H_116_N_13_NO_34_ [M + H]^+^: 1790.8; found 1790.8.

### 4.4. Trivalent GalNAc Cluster (**8**)

Compound **7** was dissolved in aqueous 0.01% TFA and placed in 55 °C oven. After 20 min, HRMS (ESI-TOF) indicated the complete hydrolysis of the acetal to aldehyde. The reaction mixture was evaporated to dryness. The obtained **8** was used as such in the conjugation. MS-ESI (*m*/*z*) calc. for C_75_H_105_N_13_NaO_33_ [M + Na]^+^: 1738.7; found 1738.6.

### 4.5. Synthesis of Conjugates **C1**–**C4** Using N-(methoxy)oxazolidine Ligation

**ON1**-**ON4** (100 nmol) and the conjugate molecules (**P1**, **P2**, **PNA1** and **8**, 2–8 equiv, cf. Table 3) in a mixture 2 mol L^−1^ LiCl in DMSO/AcOH (20 µL, 3:1, *v*/*v*) were incubated for one hour at 55 °C. The reaction mixtures were quenched by adding NaOH (0.12 M) to H_2_O/MeCN (1:1, *v*/*v*) and subjected them to RP HPLC (Figure 4). The product fractions were collected and lyophilized. Conjugate **C3^Ac^** was dissolved in concentrated aq. ammonia. The mixture was incubated for 3 h in room temperature and evaporated to dryness to yield **C3**. The yields of the isolated conjugates (Table 3) were determined from the UV absorbance at 260 nm using the molar absorptivity of the corresponding nucleobases. The authenticity of the products was verified by MS (ESI-TOF).

## Figures and Tables

**Table 1:** Hydrolysis rates of the **U^NOMe^** conjugates at 37 °C.

Entry	Conjugate ^a^	pH ^b^	t_0.5_ (h)	ON Released at Equilibrium (%)
**1 ^c^**	C1*	4	5.80 ± 0.52	95.2 ± 2.1
**2 ^c^**	C1*	5	41.7 ± 2.3	95.8 ± 1.6
**3 ^c^**	C1*	6	222 ± 20	quant.
**4**	**C1**	4	1.53 ± 0.25	91.5 ± 5.1
**5**	**C1**	5	7.17 ± 0.77	89.7 ± 2.9
**6**	**C1**	6	37.3 ± 3.3	95.7 ± 3.4
**7**	**C2**	5	4.41 ± 0.17	59.2 ± 0.6
**8**	**C3**	5	10.2 ± 0.81	94.8 ± 2.4
**9**	**C4**	5	7.40 ± 1.03	63.9 ± 2.5

^a^ 10 µM initial concentration. ^b^ 100 mM NaOAc/AcOH. ^c^ Previously published data [36].

**Table 2:** Formation and decay of *N*-(methoxy)oxazolidines between 2′-deoxy-2′-(*N*-methoxyamino) uridine and small molecular aldehydes.

Entry	R ^b^	pH	t_0.5_ Decay (h) ^c^	Equilibrium Constant *K* (l mol^−1^) ^c^	Equilibrium Yield (%) ^c^
1 ^a^	BzNHCH_2_	4	16.1 ± 0.7	4958 ± 50	82
2 ^a^	“	5	75.5 ± 16.3		
3 ^a^	“	6	n/a		
4	BzNHCH_2_CH_2_	4	5.28 ± 0.58	2398 ± 425	75
5	“	5	28.5 ± 2.2		
6	“	6	310 ± 34		

^a^ Previously published data [37]. ^b^ Cf. R in Figure 1. ^c^ According to pseudo first-order rate law. ^d^ Acquired by mixing **1** (5 mM) and aldehyde (5 mM) at pH 4.

**Table 3:** Data of the *N*-(methoxy)oxazolidine ligation to obtain conjugates **C1**–**C4**.

Conjugate	Aldehyde	Aldehyde Excess (Equiv)	Isolated Yield ^d^	Observed Molecular Mass	Calculated Molecular Mass
**C1**	**P1**	8	25%	9999.4 ^a^	9999.8
**C2**	**P2**	8	19%	9006.2 ^b^	9006.3
**C3**	**8**	5	47%	8851.1 ^c^	8852.7
**C4**	**PNA1**	2	43%	8860.4 ^c^	8859.8

Observed molecular masses were calculated from the most intensive isotope at ^a^ [(M–10H)/10)]^10^, ^b^ [(M–6H)/6]^6−^, and ^c^ [(M-4H)/4]^4−^. ^d^ Yields were determined from the UV absorbance at 260 nm using the molar absorptivity of the corresponding nucleobases.

## Data Availability

The primary data presented in this study are available on request from the corresponding author.

## Supplementary Materials

The following are available online: small-molecule syntheses (*N*-Bz-3-amino-1,1-diethoxypropane, *N*-Bz-β-Ala-H, *N*-Fmoc-3-amino-1,1-diethoxypropane); NMR (^1^H and ^13^C) spectra of compounds **5**, **6**, and **7**; synthesis of **ON1**, **ON2**, **ON3**, **ON4**, and their mass spectra and RP HPLC chromatograms after purification; synthesis of oxazolidine β-Ala-H solid support **3**; synthesis of **P1**, **P2**, and **PNA1** and their mass spectra and RP HPLC chromatograms after purification; mass spectra for conjugates **C1**, **C2**, **C3^Ac^**, **C3**, and **C4**, small molecule model: studying the reversible *N*-(methoxy)oxazolidine formation between **1** and *N*-Bz-β-Ala-H including representative RP HPLC profile, kinetic profiles, and NMR (^1^H and ^13^C) characterization of the ligation products; determining hydrolysis rates of the conjugates **C1**, **C2**, **C3**, and **C4** including kinetic profiles. References [56,57,58] are cited in the supplementary materials.
