# Supplementary material for: Expanding the Scope of the Cleavable N-(Methoxy)oxazolidine Linker for the Synthesis of Oligonucleotide Conjugates
Source: Molecules. 2021 Jan 18;26(2):490. doi: 10.3390/molecules26020490 (PMC7838870; doi:10.3390/molecules26020490)
Supplement: Supplementary file 1 [file molecules-26-00490-s001.pdf]

Supporting information to:

Expanding the scope of the cleavable N-(methoxy)oxazolidine linker for the synthesis of oligonucleotide conjugates

Aapo Aho, Antti Äärelä, Heidi Korhonen, Pasi Virta* (pamavi@utu.fi)

Department of Chemistry, University of Turku, 20014 Turku, Finland

Contents

1. Small molecule syntheses 3

1.1. *N*-Bz-3-amino-1,1-diethoxypropane 3

1.2. *N*-Bz-β-Ala-H 3

1.3. *N*-Fmoc-3-amino-1,1-diethoxypropane 3

Figure S1. ^{1}H NMR (600 MHz, $CDCl_3$) spectrum of *N*-Fmoc-3-amino-1,1-diethoxypropane. 4

Figure S2. ^{13}C NMR (125 MHz, $CDCl_3$) spectrum of *N*-Fmoc-3-amino-1,1-diethoxypropane. 5

1.4. *N*-Fmoc-β-Ala-H 5

2. Synthesis of U^{NOMe}-oligonucleotides ON1, ON2, ON3, and ON4 6

Figure S3. RP HPLC profiles of synthesized U^{NOMe}-oligonucleotides ON1, ON2, ON3, and ON4 after purification. 7

Figure S4. Mass spectra of the U^{NOMe}-oligonucleotides ON1, ON2, ON3, and ON4. 7

3. Oxazolidine ß-Ala-H solid support for peptide/PNA aldehyde synthesis (3) 8

4. Synthesis of β-Ala-H peptide aldehydes P1 and P2: 8

Figure S5. RP HPLC profiles (C18, 250 × 10 mm, 5 μm)) of the β-Ala-H peptide aldehydes (P1 and P2) and PNA aldehyde (PNA1) after purification. 9

5. Synthesis of PNA1 9

6. Conjugation of UNOMe-ASOs and β-Ala-H conjugate groups via N-(methoxy)oxazolidine 10

Figure S7. Mass spectra of the U^{NOMe}-conjugates C1, C2, $C3^{Ac}$, C3, and C4. 10

7. Small molecule model 11

Figure S9. Reversible N-(methoxy)oxazolidine formation between 1 and *N*-Bz-Ala-H. 11

Figure S10. Hydrolysis reaction profiles for the major ligation product of 1 and *N*-Bz-β-Ala-H 12

8. Characterization of *N*-Bz-β-Ala-H ligation products 12

Figure S11. ^{1}H NMR (600 MHz, CD_3CN) spectrum of the *N*-Bz-β-Ala-H major ligation product. 13

Figure S12. ^{13}C NMR (125 MHz, CD_3CN) spectrum of the *N*-Bz-β-Ala-H major ligation product. 14

Figure S13. ^{1}H NMR (600 MHz, CD_3CN) spectrum of the *N*-Bz-β-Ala-H minor ligation product. 14

Figure S14. ^{13}C NMR (125 MHz, CD_3CN) spectrum of the *N*-Bz-β-Ala-H minor ligation product. 15

9. Determining hydrolysis rates of ASO-U^{NOMe} conjugates C1, C2, C3, and C4 15

Figure S15. Hydrolysis reaction profiles of U^{NOMe}-conjugates C1, C2, C3, and C4. 16

10. NMR spectra of compounds 5, 6, and 7 17

Figure S16. ^{1}H NMR (600 MHz, $CDCl_3$) spectrum of compound 5. 17

Figure S17. ^{13}C NMR (125MHz, $CDCl_3$) spectrum of compound 5. 17

Figure S18. ^{1}H NMR (600 MHz, $CDCl_3$) spectrum of compound 6. 18

Figure S19. ^{13}C NMR (125MHz, $CDCl_3$) spectrum of compound 6.. 18

Figure S20. ^{1}H NMR (600MHz, MeOD-$CDCl_3$, 1:9, *v*/*v*) spectrum of compound 7. 19

Figure S21. ^{13}C NMR (150MHz, MeOD-$CDCl_3$, 1:9, *v*/*v*) spectrum of compound 7. 20

1. Small molecule syntheses

1.1. *N*-Bz-3-amino-1,1-diethoxypropane

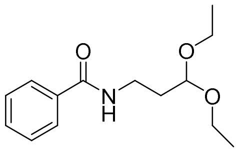

Benzoyl chloride (1.6 mL, 1.9 g, 14 mmol) was added slowly to a mixture of 3,3-diethoxypropan-1-amine (Across Organics, 2.0 g, 14 mmol) and anhydrous pyridine (2.0 mL, 25 mmol) in DCM (25 mL) at 0°C, and the mixture was then allowed to warm up to room temperature. The mixture was stirred overnight, saturated aqueous $NaHCO_3$ (70 mL) was added, and the product was extracted with DCM (4 × 50 mL). The combined organic phases were washed with H_2O (3 × 50 mL) and brine (50 mL), dried with Na_2SO_4, filtered, and evaporated to dryness. The residue was purified silica gel chromatographically (30-100% EtOAc in hexane). The product (1.4 g, 40%) was amorphic white to transparent substance. Characterization (^{1}H NMR, ^{13}C NMR and HRMS) matched with the published data[1].

1.2. *N*-Bz-β-Ala-H

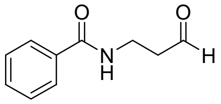

N-Bz-3-amino-1,1-diethoxypropane (0.63 g, 2.5 mmol) was dissolved in THF (10 mL) and the solution was cooled down to 0 °C on ice bath. Aqueous 10% HCl (5 mL) was slowly added to the solution while stirring vigorously with a magnetic stirrer. The ice bath was removed and the mixture was allowed to warm up to room temperature. The reaction was quenched by dropwise addition of aqueous NaOH (1 M) until pH reached 7. The mixture was evaporated to dryness. The residue was suspended in MeCN (7 mL), filtered, and the filtrate was evaporated to dryness. The residue was purified by RP HPLC using a linear gradient (10-56% aq MeCN) over 20 min. After lyophilization, the product (0.16 g, 36%) was obtained as a white powder. Characterization (^{1}H NMR, ^{13}C NMR and HR MS) matched with the published data[2].

1.3. *N*-Fmoc-3-amino-1,1-diethoxypropane

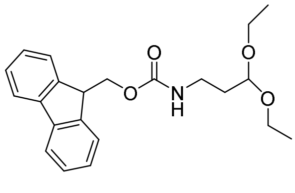

9-Fluorenylmethoxycarbonyl chloride (9.3 g, 41 mmol) was added to a mixture of K_2CO_3 (5.0 g, 41 mmol) and 3,3-diethoxypropan-1-amine (4.4 g, 30 mmol) in dioxane/water (1:1, *v/v,* 200 mL). The mixture was stirred for one hour at room temperature, neutralized with NH_4Cl and evaporated to dryness. The residue was extracted twice with EtOAc (200 mL, 100 mL). The organic phase was washed with H_2O (70 mL) and brine (70 mL), dried with Na_2SO_4, filtered, and evaporated to dryness. The crude product was purified silica gel chromatographically (20% EtOAc in hexane). The product (8.6 g, 77%) was obtained as a yellow transparent oil, which crystallized after storing overnight in desiccator. **^{1}H**

NMR δH (500 MHz, $CDCl_3$): 7.79 (2H, d, *J* = 7.5 Hz), 7.62 (2H, d, *J* = 7.5 Hz), 7.42 (2H, t, *J* = 7.5 Hz), 7.33 (2H, t, *J* = 7.5 Hz), 5.36 (1H, b), 4.60 (1H, t, *J* = 5.0 Hz), 4.41 (2H, d, *J* = 7.0 Hz), 4.25 (1H, t, *J* = 7.0 Hz), 3.71 (2H, m), 3.54 (2H, m), 3.35 (2H, m), 1.87, (2H, m), 1.26 (6H, t, *J* = 7.15 Hz); **^{13}C NMR** δC (125 MHz, $CDCl_3$): 156.4, 144.1, 141.4, 127.7, 125.1, 120.0, 66.6, 61.8, 47.3, 37.2, 33.3, 15.4; **HRMS-ESI** (*m*/*z*) calc. for $C_{22}H_{27}NNaO_4$ [M + Na]$^+$: 392.1838; Found: 392.1836.

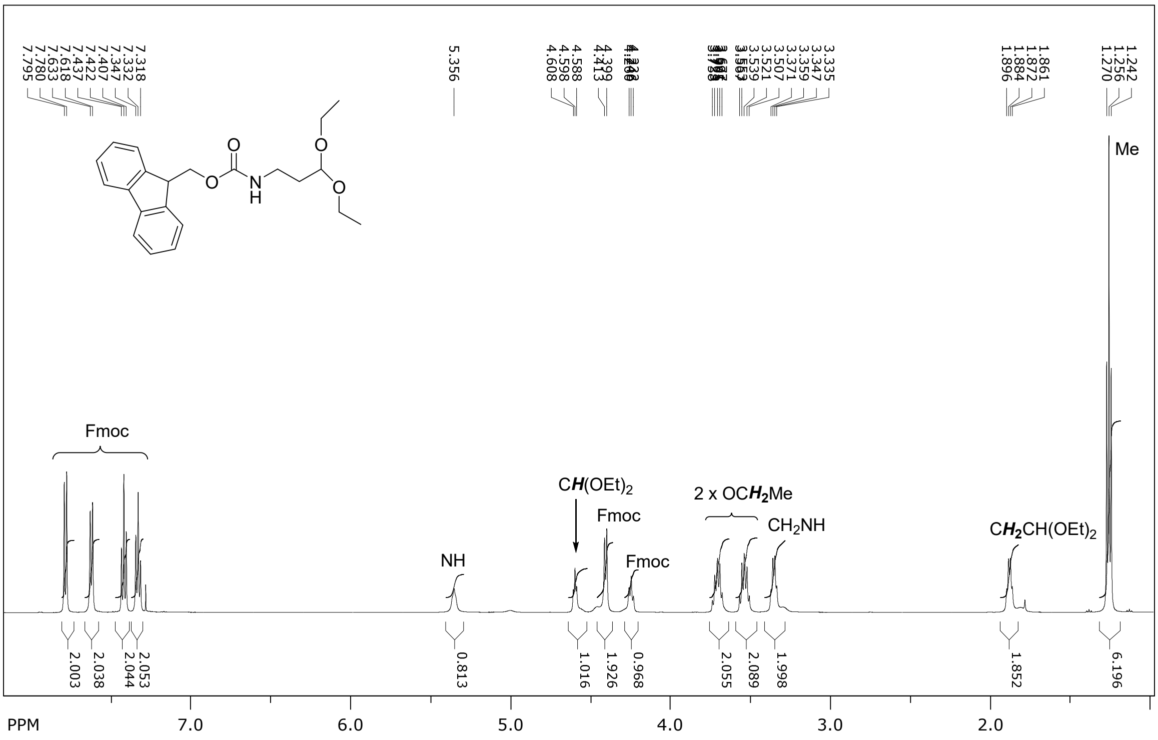

Figure S1. ^{1}H NMR (600 MHz, $CDCl_3$) spectrum of *N*-Fmoc-3-amino-1,1-diethoxypropane.

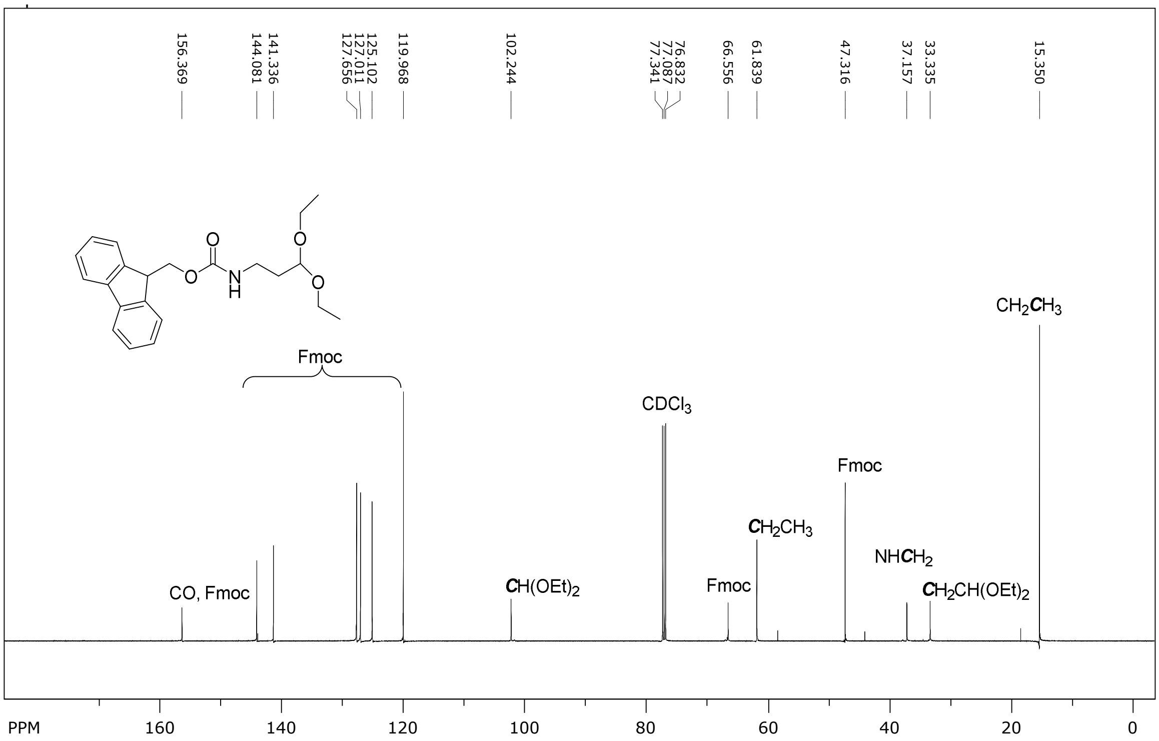

Figure S2. ^{13}C NMR (125 MHz, $CDCl_3$) spectrum of *N*-Fmoc-3-amino-1,1-diethoxypropane.

1.4. *N*-Fmoc-β-Ala-H

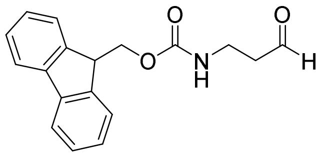

A solution of *N*-Fmoc-3-amino-1,1-diethoxypropane (2.20 g, 5.95 mmol) in THF (50 mL) was cooled down to 0 °C using ice bath. Aqueous 10% HCl (10 mL) was added dropwise to the solution while stirring vigorously. After addition, the mixture was stirred for 15 min and the reaction was quenched by a dropwise addition of sat. aq. Na_2HCO_3 until the solution was slightly basic. The product was extracted twice with EtOAc (250 mL, 70 mL). The organic phase was washed with $NaHCO_3$ (2 × 15 mL), dried with Na_2SO_4, filtered, and evaporated to dryness. The product (1.5 g, 88%) was used as such in the next step. Characterization (^1H NMR, ^{13}C NMR and HRMS) matched with the published data[3].

2. Synthesis of U^{NOMe}-oligonucleotides ON1, ON2, ON3, and ON4

U^{NOMe} =

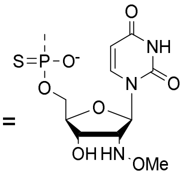

Bold letters are 2´-*O*-methylribonucleotides, underlined are 2´-*O*-(2-methoxyethyl)ribonucleotides, and the rest are 2´-deoxyribonucleotides

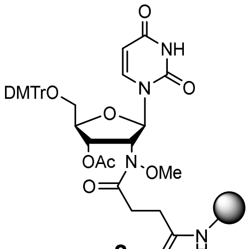

2

ON1: CUAGUAUGAAAGAGAGACAUUGU ^{NOMe}
ON2: UCACUUUCAUAAUGCUGG **U**^{NOMe}
ON3: TCCATTTATTAGTCT AGGAA**U**^{NOMe}
ON4: GCGTGGTCACACGCTTT **U**^{NOMe}

U^{NOMe}-oligonucleotides **ON1**, **ON2**, **ON3**, and **ON4** were synthesized on 1.0 µmol scale using an automatic DNA/RNA-synthesizer, commercially available phosphoramidite building blocks and solid support **2**[4]. Benzylthiotetrazol was used as an activator. For the sulfurization 3-phenyl-1,2,4-dithiazoline-5-one with 150 s contact time was used. After the chain assembly, the solid-supported oligonucleotides were exposed to concentrated aqueous ammonia for 16 h at 55 °C. The suspensions were filtered, and filtrates were evaporated to dryness. The residues were dissolved in water and purified by RP HPLC (C18, 250 × 10 mm 5 µm) with a linear gradients of 5-45% MeCN in aquous 50 mM triethylammonium acetate (pH 7) over 25 min. After lyophilization, the oligonucleotides were characterized by MS (ESI-TOF) and quantified UV spectrophotometrically (at 260 nm).

Oligonucleotide	Isolated Yield [%]	Observed Molecular Mass	Calculated Molecular Mass
**ON1**	33	[a]8109.6	8110.5
**ON2**	32	[b]7477.4	7478.4
**ON3**	29	[b]7531.0	7531.3
**ON4**	40	[b]5511.7	5512.6

Observed molecular masses were calculated from the most intensive isotope at [a]$[(M–6H)/6)]^{6-}$, [b]$[(M–4H)/4]^{4-}$.

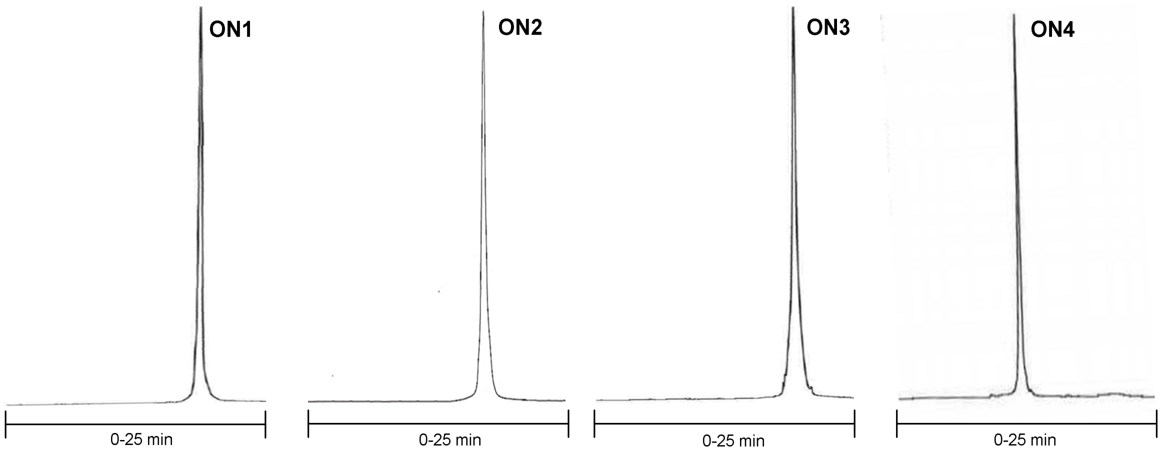

Figure S3. RP HPLC profiles of synthesized **U**^{NOMe}-oligonucleotides **ON1**, **ON2**, **ON3**, and **ON4** after purification.

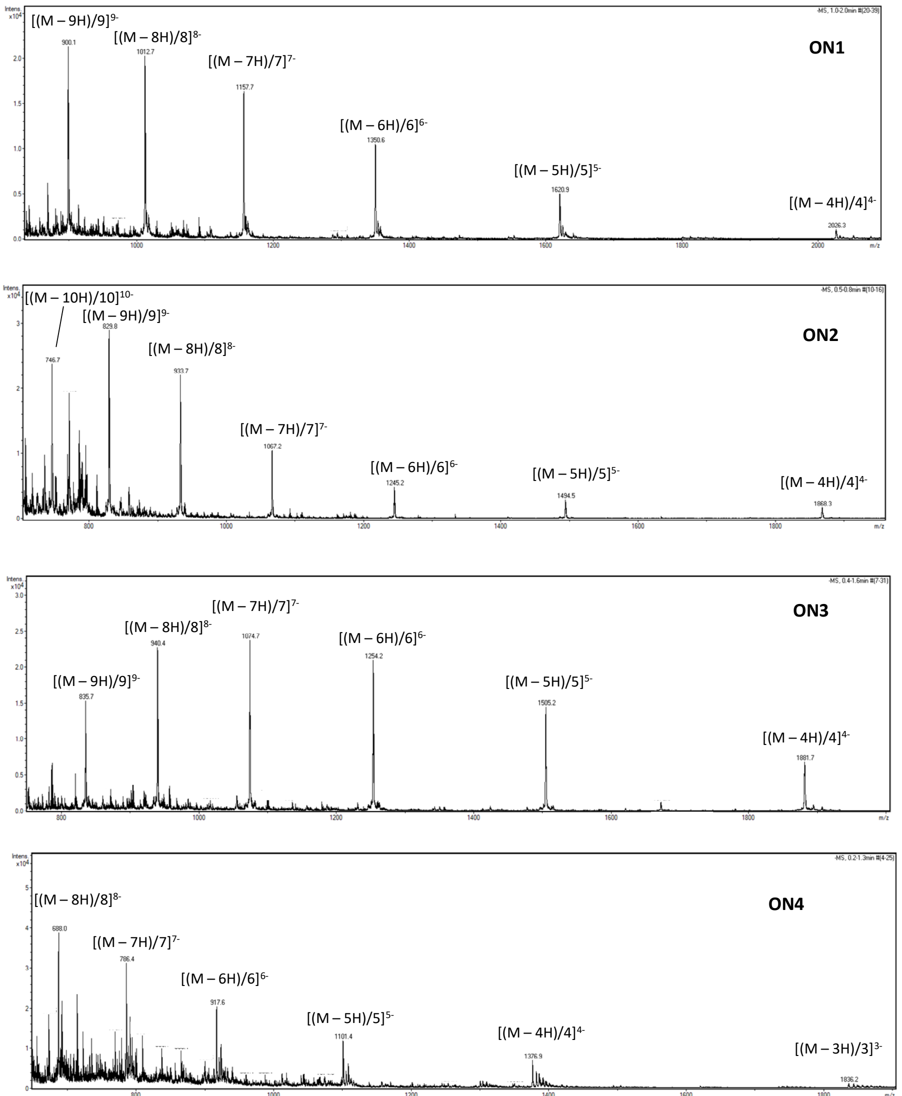

Figure S4. Mass spectra of the **U**^{NOMe}-oligonucleotides **ON1**, **ON2**, **ON3**, and **ON4**.

3. Oxazolidine ß-Ala-H solid support for peptide/PNA aldehyde synthesis (3)

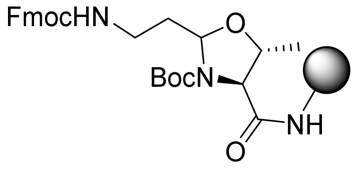

3

H-RINK amide ChemMatrix resin was elongated with Gly and Thr as described before[4]. The ThrGly-RINK resin (1.0 g, loading of 286 µmol g^{-1}) was swelled by adding DIPEA/DMF (1/99, *v/v,* 12 mL). Fmoc-β-Ala-H (680 mg, 2.30 mmol) was dissolved in DIPEA/DMF (1/99, *v/v,* 3 mL) and then mixed with the swelled resin. The suspension was placed on an oil bath (60 °C) and gently mixed using a magnetic stirrer. After 2 h, the resin was filtered and washed with DMF, MeOH, and DCM. The resin was exposed to piperidine/DMF (20/80, *v/v*) for 10 min, filtered, and washed with DMF, MeOH, and DCM (20 mL each). The resin was swelled by adding dry THF (10 mL) and di-*tert*-butyl dicarbonate (0.50 g, 2.29 mmol) and *N*-methylmorpholine (251 µl, 2.29 mmol) in dry THF (5 mL) were added. The obtained suspension was placed in an oil bath (50 °C) and mixed using a magnetic stirrer. After 3 h, the resin **3** was filtered, washed with DMF, MeOH, and DCM (20 mL each) and dried under vacuum. The loading of 158 µmol g^{-1} was determined using Fmoc cleavage assay.

4. Synthesis of β-Ala-H peptide aldehydes P1 and P2:

P1: H_2N-Val-Pro-Thr-Ile-Val-Met-Val-Asp-Ala-Tyr-Lys-Pro-Thr-Lys-(AEEA)$_2$-ß-Ala-H

P2: H_2N-Pro-Trp-Val-Pro-Ser-Trp-Met-Pro-Pro-Arg-His-Thr-ß-Ala-H

Peptide aldehydes **P1** and **P2** were synthesized on 10 µmol scale by using an automated microwave-assisted peptide synthesizer, standard Fmoc/tBu-protected amino acids and the oxazolidine β-Ala-H solid support **3**. DIC/oxyma was used as an activator. Coupling times of 4 min at 90 °C were used (exceptions: Arg residue a double coupling of 2 min at 90 °C. His residue: a coupling of time of 10 min at 50 °C). Piperidine in DMF (1:4, *v/v*) was used for the Fmoc deprotection (1 min at 90 °C). After the chain assembly, the oxazolidine-protected peptide aldeyhdes were cleaved from the support with a mixture of anisol and TFA (1:9, *v/v*, 1 mL) for **P1** and a mixture of anisol, EDT, thioanisol and TFA (2:3:5:90, *v/v/v/v*, 1 mL) for **P2**. After cleavage, the peptides were precipitated in cold diethyl ether (10 mL) and centrifuged. The pellets were dissolved in aq. 0.01 % TFA (1 mL) and purified by RP HPLC (conditions in **figure S5**). Authenticity of the purified peptide aldehydes was verified by MS (ESI-TOF). Isolated yields were determined from the UV absorbance at 280 nm.

Peptide Aldehyde	Isolated Yield [%]	Observed Molecular Mass	Calculated Molecular Mass
**P1**	27	1907.1[a]	1907.3
**P2**	12	1545.8[a]	1545.8

Observed molecular masses were calculated from the most intensive isotope at [a][(M+2H)/2]$^{2+}$.

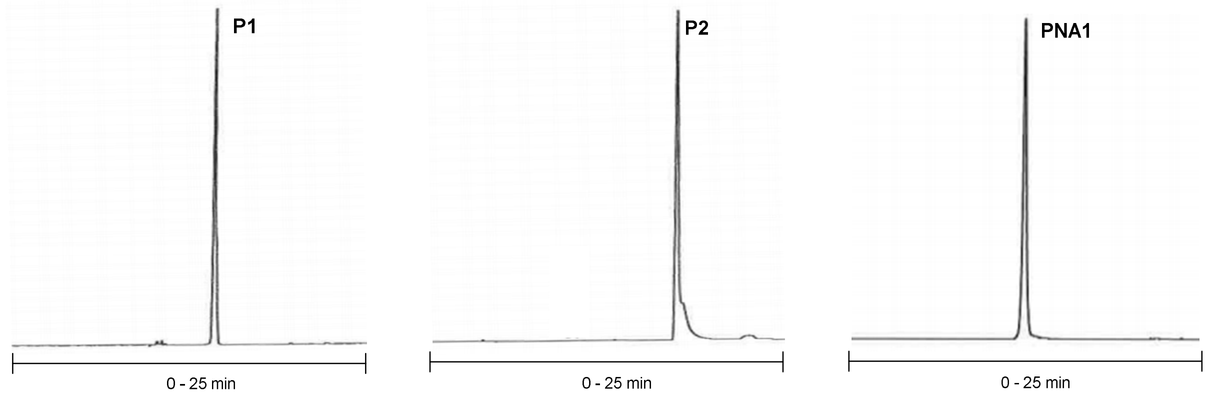

Figure S5. RP HPLC profiles (C18, 250 × 10 mm, 5 µm)) of the β-Ala-H peptide aldehydes (**P1** and **P2**) and PNA aldehyde (**PNA1**) after purification. Linear gradients of 0-90% MeCN (**P1** and **P2**) and 0-60% (**PNA1**) in aq. TFA (0.1%) over 25 min, flow rate of 2.5 mL min^{-1}, and detection wavelengths of 280 nm (**P1** and **P2**) and 260 nm (**PNA1**) were employed.

5. Synthesis of PNA1

β-Ala-H PNA aldehyde **PNA1** was synthesized on 10 µmol scale by using an automated peptide synthesizer, standard Fmoc/Bhoc-protected PNA-building blocks and the oxazolidine β-Ala-H support **3**. PyBOP with DIPEA was used as an activator. Coupling times of 35 min at rt were used, followed by 5 min of capping with Ac_2O (2%) in a mixture of DMF and pyridine (1:1, *v*/*v*). Piperidine in DMF (1:4, *v*/*v*) was used for the Fmoc deprotection (10 min in rt). After the chain assembly, the oxazolidine protected PNA was cleaved from the support using a mixture of anisole and TFA (1:9, *v*/*v*, 1 mL). After cleavage, the PNA was precipitated in cold diethyl ether (10 mL) and centrifuged. The pellet was dissolved in aq. TFA (0.01%) and purified by RP HPLC (conditions in **Figure S5**). Authenticity of **PNA1** was verified by MS (ESI-TOF). Isolated yield was determined from the UV absorbance at 260 nm.

**Peptide Aldehyde**	**Isolated Yield [%]**	**Observed Molecular Mass**	**Calculated Molecular Mass**
**PNA1**	7.1	3364.7[a]	3365.3 (as aldehyde)
		3346.4[a]	3346.2 (as cyclic imine)

Observed molecular masses were calculated from the most intensive isotope at $^{a}[(M+2H)/2]^{2+}$.

6. Conjugation of UNOMe-ASOs and β-Ala-H conjugate groups via N-(methoxy)oxazolidine

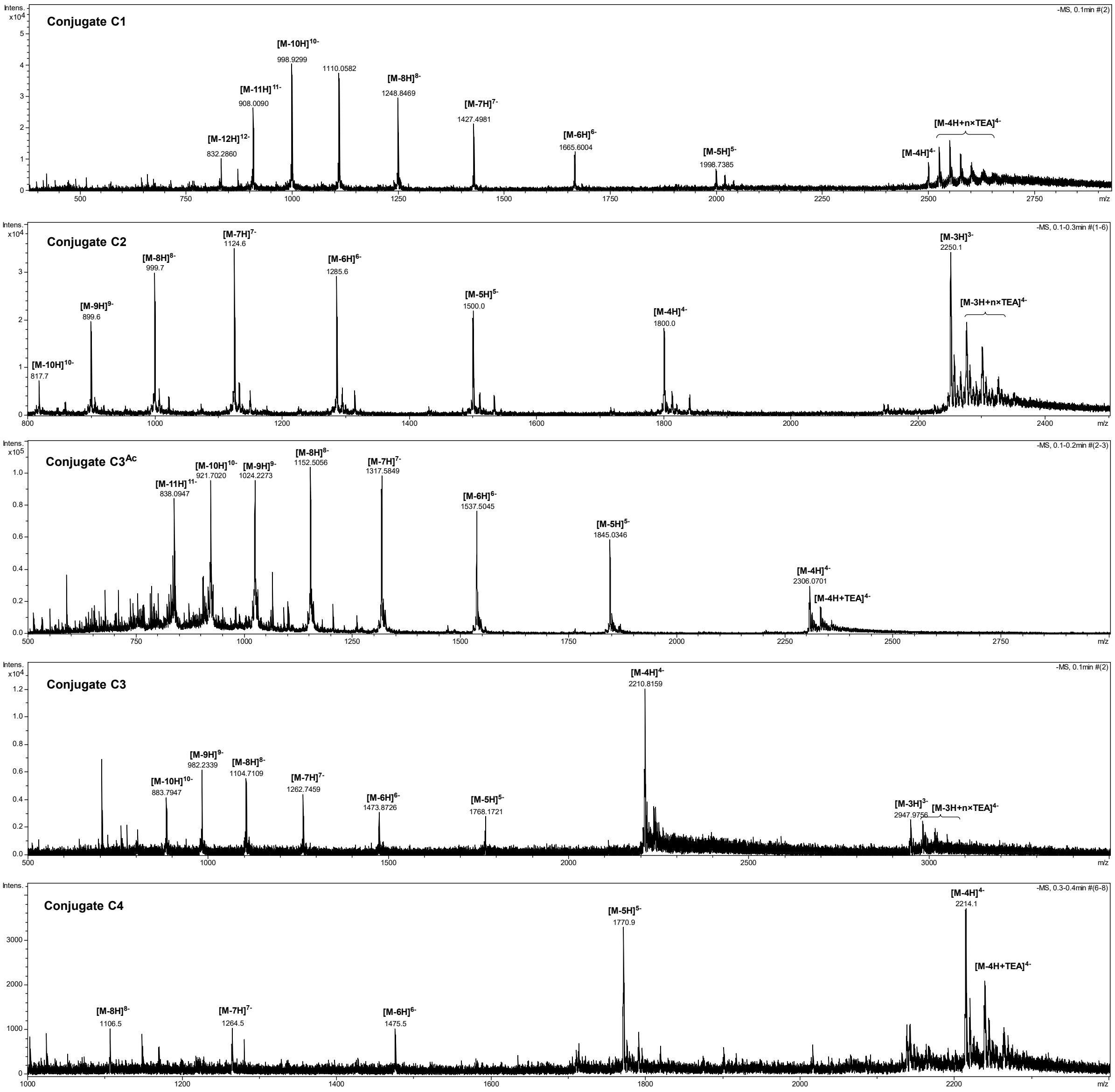

Figure S7. Mass spectra of the U^NOMe-conjugates **C1**, **C2**, **C3**^Ac, **C3**, and **C4**.

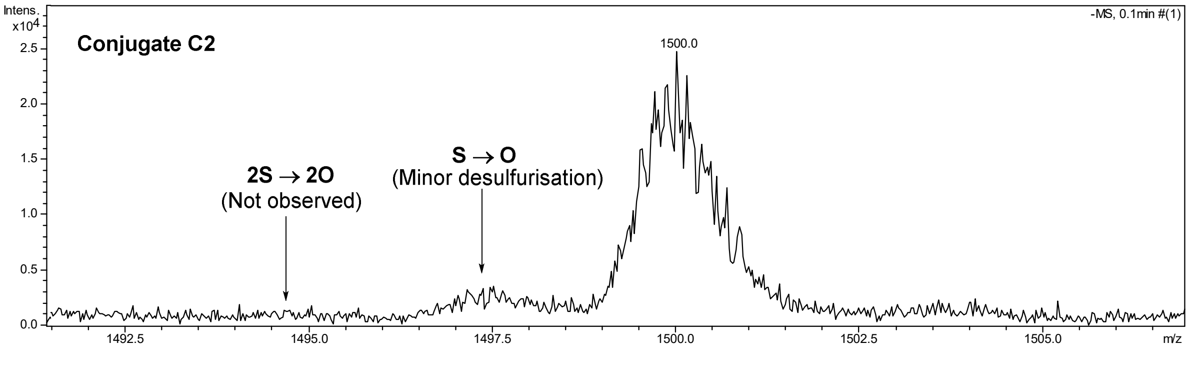

Figure S8. A representative mass spectrum of desulfurization in U^NOMe-phosphorothioate conjugates.

7. Small molecule model

Buffer solutions used in small molecule model studies:

0.1 M AcOH, pH 4 (adjusted using NaOH), $I = 0.1$ L mol^{-1} (adjusted using NaCl)
0.1 M AcOH, pH 5 (adjusted using NaOH), $I = 0.1$ L mol^{-1} (adjusted using NaCl)
0.1 M MES, pH 6 (adjusted using NaOH), $I = 0.1$ L mol^{-1} (adjusted using NaCl)
0.2 M triethylammonium acetate (TEAA), pH 7

An aqueous mixture containing *N*-Bz-β-Ala-H (5 mM) and the appropriate buffer (200 uL)[3] was incubated for 30 min to ensure total solubilization of the aldehyde. Nucleoside analog **1** (5 mM) was added to the mixture, and the reaction vessel was capped, vortexed and placed in room temperature (22.0 ± 1.5 °C). Samples (1 µL) were taken at adequate intervals and quenched with TEAA buffer (100 µL, pH 7). The quenched samples were analyzed by RP HPLC (conditions in **Figure S9b**) Two ligation products, were formed (**Figure S9**), which were characterized (^{1}H NMR, ^{13}C NMR, HRMS) as stereoisomers of *N*-(methoxy)oxazolidine condensation product between **1** and *N*-Bz-β-Ala-H. The reactant concentrations were determined by comparing the reactant peak absorbances to the total absorbances of reactive species (using molar absorptivities at 260 nm (ε_{260}) of ε_{260}(**1**) = 9660 M^{-1}cm^{-1}, ε_{260}(*N*-Bz-β-Ala-H)[5] = 970 M^{-1} cm^{-1}, and ε_{260}(ligation product) = ε_{260}(**1**) + ε_{260}(*N*-Bz-β-Ala-H) = 10630 M^{-1} cm^{-1}). Equilibrium constant K = (2398 ± 425) M^{-1} was obtained for the total ligation product formation. (**Figure S9a**)

To obtain the hydrolysis half-lifes ($t_{0.5}$), the isolated major isomer (1 mM) was dissolved in aq. buffer (200 µL) pH 4, 5, or 6 and the reaction was monitored by RP HPLC as described above. The half-life for hydrolysis was determined according to the 1st order reaction rate law (**Figure S10**).

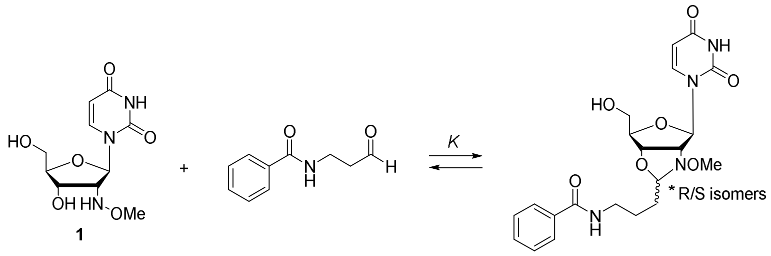

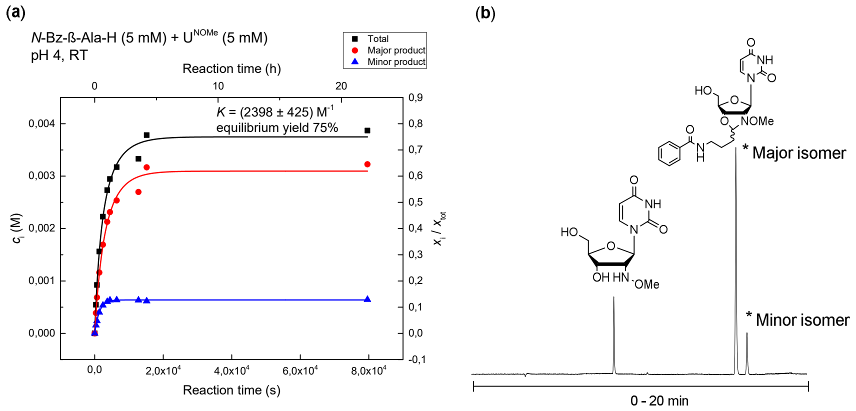

Figure S9. Reversible *N*-(methoxy)oxazolidine formation between **1** and *N*-Bz-Ala-H. (**a**) Reaction profile for formation of *N*-(methoxy)oxazolidine acquired by mixing **1** (5 mM) and *N*-Bz-Ala-H (5 mM) in aq. buffer (pH 4) at rt. Equilibrium constant K = (2398 ± 425) M^{-1} was obtained. (**b**) RP HPLC (C18

column, 250 × 4.6 mm, 5 μm) profile at $t = 22$ h showing **1** and *N*-(methoxy)oxazolidine ligation products (major and minor stereoisomers). A linear gradient of MeCN (0-60% in 20 min) over aq. TEAA (pH 7), a flow rate of 1.0 mL min^{-1}, and a detection wavelength of 260 nm were employed.

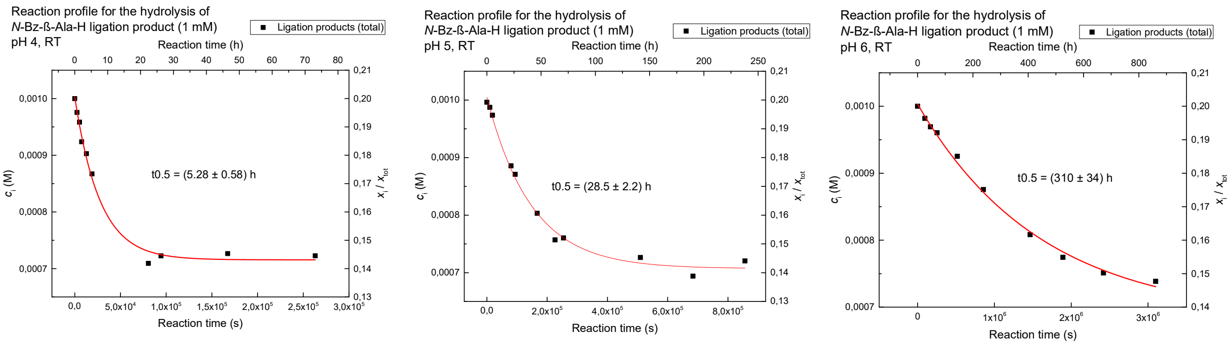

Figure S10. Hydrolysis reaction profiles for the major ligation product of **1** and *N*-Bz-β-Ala-H obtained by dissolving the major isomer (1 mM) in aq. buffer pH 4, 5 or 6 at rt.

8. Characterization of *N*-Bz-β-Ala-H ligation products

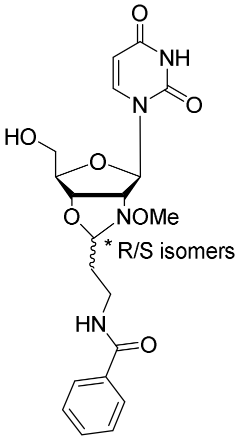

N-Bz-β-Ala-H major ligation product

^{1}H NMR δ_H (600 MHz, CD_3CN): 7.82 (2H, d, $J = 7.3$ Hz, Bz), 7.64 (1H, d, $J_{H6,H5} = 8.2$ Hz, H6), 7.55 (1H, t, $J = 7.3$ Hz, Bz), 7.49 (2H, t, $J = 7.4$ Hz, Bz), 7.24 (1H, br, Bz), 5.68 (2H, m, H5, H1´), 4.70 (1H, t, $J = 3.8$ Hz, H3´), 4.66 (1H, br, CH-oxazolidine), 4.30 (1H, br, H2´), 4,02 (1H, q, $J = 3.8$ Hz, H4´), 3,75 (1H, dd, $J = 12.0$ and 3.4 Hz, H5´), 3.70 (1H, dd, $J = 12.0$ and 4.3 Hz, H5´´), 3.54 (2H, m, CH_2-β-Ala), 3.51 (3H, s, OCH_3), 2.24 and 2.13 (2 × 1H, br, CH_2-β-Ala). **^{13}C NMR** δ_C (125 MHz, CD_3CN): 167.5 (CO, Bz), 163.5 (C4), 151.1 (C2), 142.0 (C6), 135.5 (Bz), 131.8 (Bz), 129.0 (2C, Bz), 127.5 (2C, Bz), 102.9 (C5), 95.0 (CH-oxaoxolidine), 89.0 (C1´), 86.2 (C4´), 79.3 (C3´), 77.8 (C2´), 62.5 (C5´), 61.3 (OCH_3), 37.1 (β-Ala), 28.8 (β-Ala). **HRMS-ESI** (*m*/*z*) calc. for $C_{20}H_{24}N_4NaO_7$ [M + Na]$^+$: 455.1537; Found: 455.1543.

N-Bz-β-Ala-H minor ligation product

^{1}H NMR δ_H (600 MHz, CD_3CN): 7.82 (2H, d, $J = 7.1$ Hz, Bz), 7.74 (1H, d, $J_{H6,H5} = 8{,}0$ Hz, H6), 7.56 (1H, t, $J = 7.3$ Hz, Bz), 7.49 (2H, t, $J = 7.3$ Hz, Bz), 7.21 (1H, br, NH), 6.03 (1H, br, H1´), 5.67 (1H, d, $J_{H5,H6} = 8.2$ Hz,

H5), 4.79 (1H, br, CH-oxazolidine), 4.74 (1H, br, H3′), 4.22 (1H, br, H4′), 4.17 (1H, br, H2′), 3.73 (1H, dd, *J* = 12.0 and 3.2 Hz, H5′), 3.70 (1H, dd, *J* = 12.0 and 3.7 Hz, H5′′), 3.57 (5H, m, CH_2-β-Ala and OCH_3), 2.08 (2H, CH_2-β-Ala). **^{13}C NMR** δ_C (125 MHz, CD_3CN): 167.3 (CO, Bz), 163.4 (C4), 151.1 (C2), 142.0 (C6), 135.4 (Bz), 131.8 (Bz), 129.0 (2C, Bz), 127.5 (2C, Bz), 102.8 (C5), 101.1 (CH-oxazolidine), 90.5 (C1′), 86.5 (C4′), 81.7 (C3′), 77.0 (C2′), 62.3 (C5′), 62.0 (OCH_3), 36.1 (β-Ala), 32.9 (β-Ala). **HRMS-ESI** (*m*/*z*) calc. for $C_{20}H_{24}N_4NaO_7$ [M + Na]$^+$: 455.1537; Found: 455.1548.

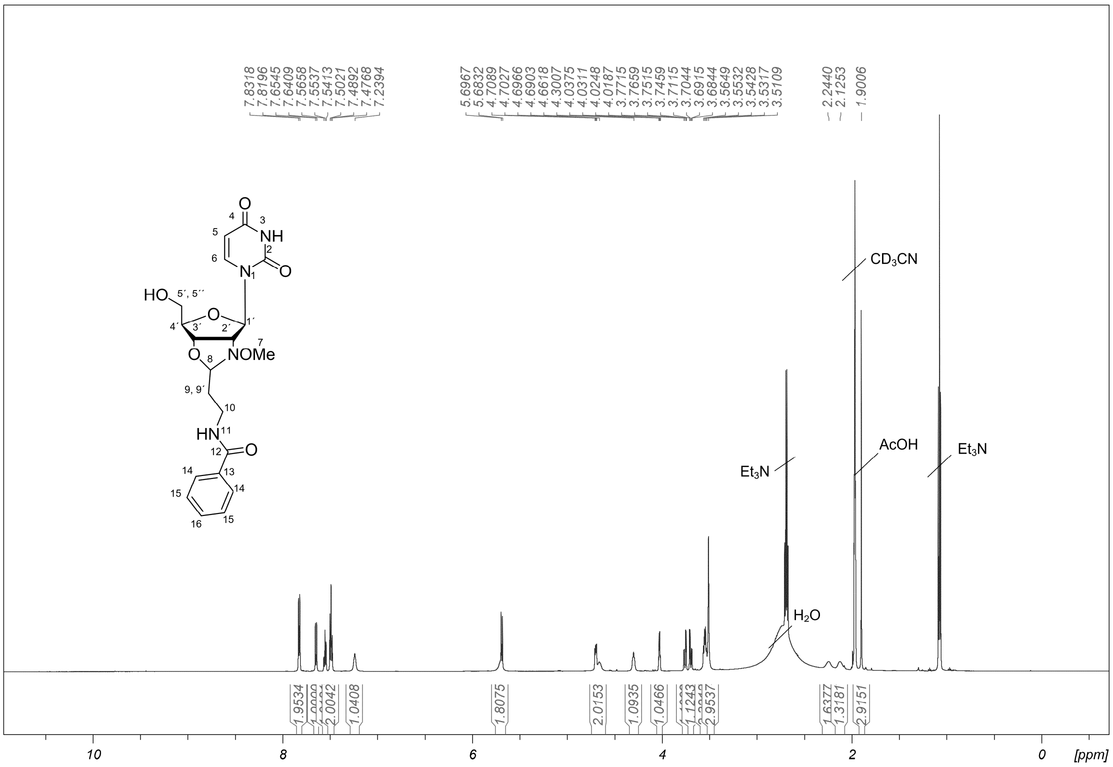

Figure S11. ^{1}H NMR (600 MHz, CD_3CN) spectrum of the *N*-Bz-β-Ala-H major ligation product.

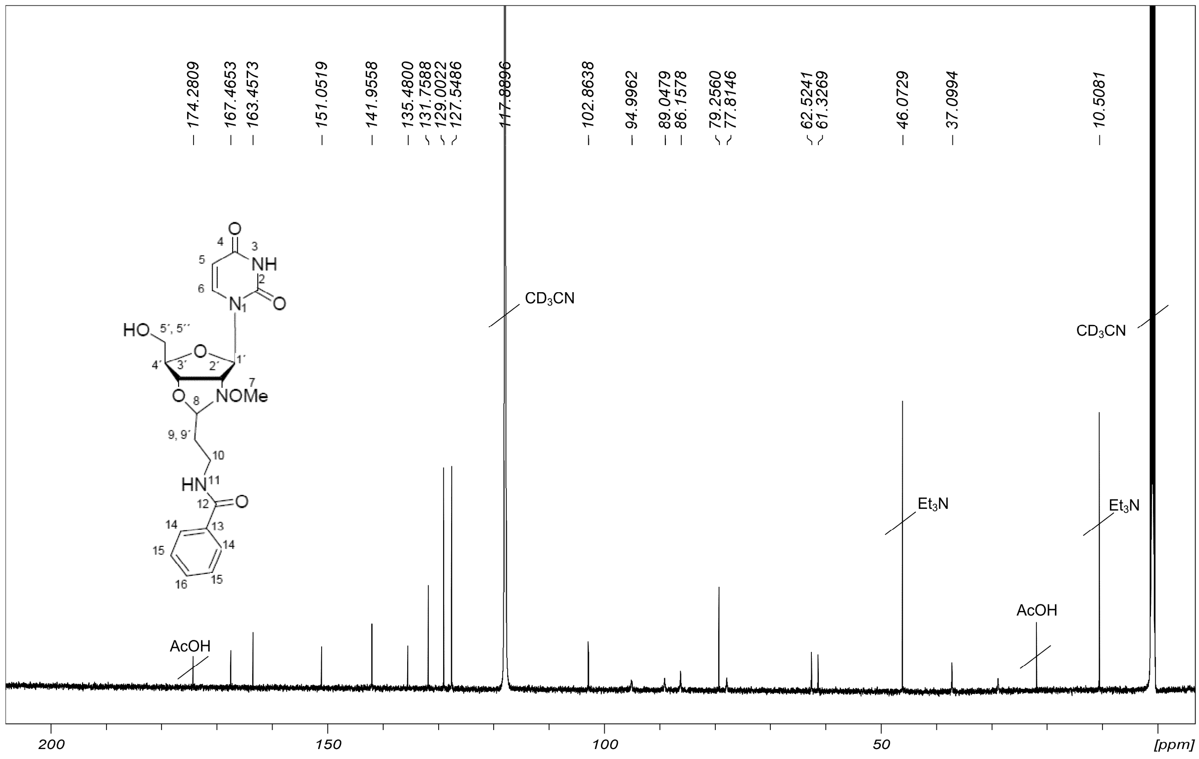

Figure S12. ^{13}C NMR (125 MHz, CD_3CN) spectrum of the *N*-Bz-β-Ala-H major ligation product.

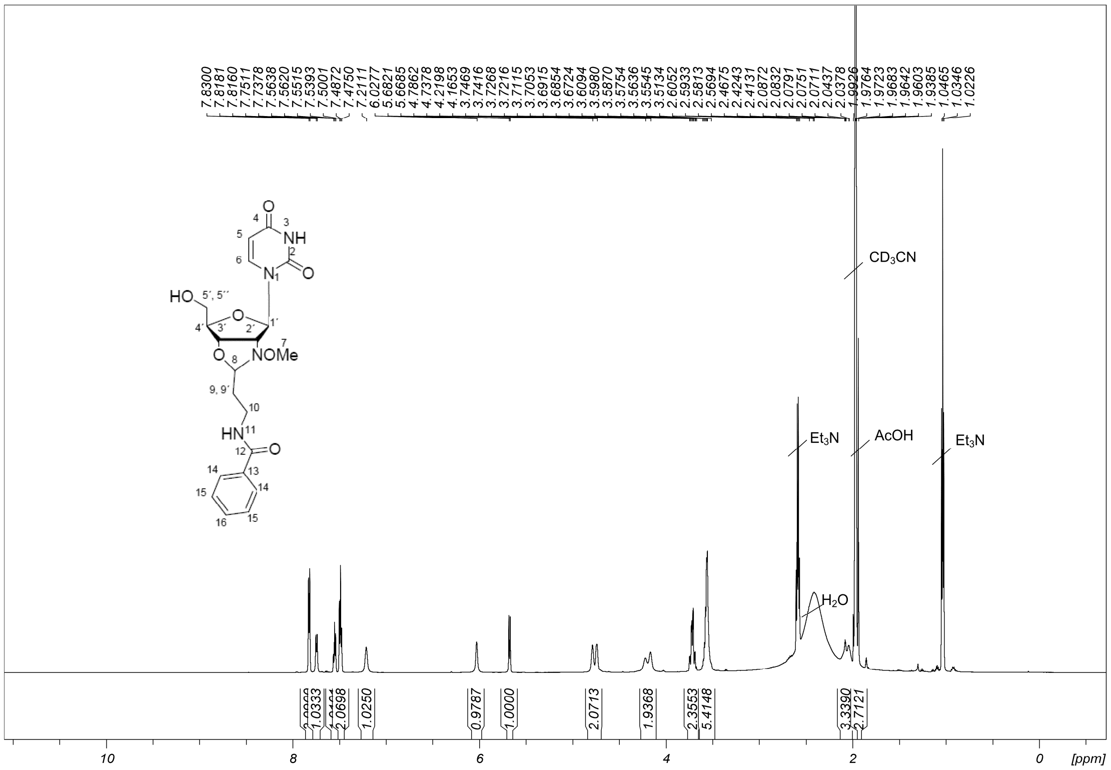

Figure S13. ^{1}H NMR (600 MHz, CD_3CN) spectrum of the *N*-Bz-β-Ala-H minor ligation product.

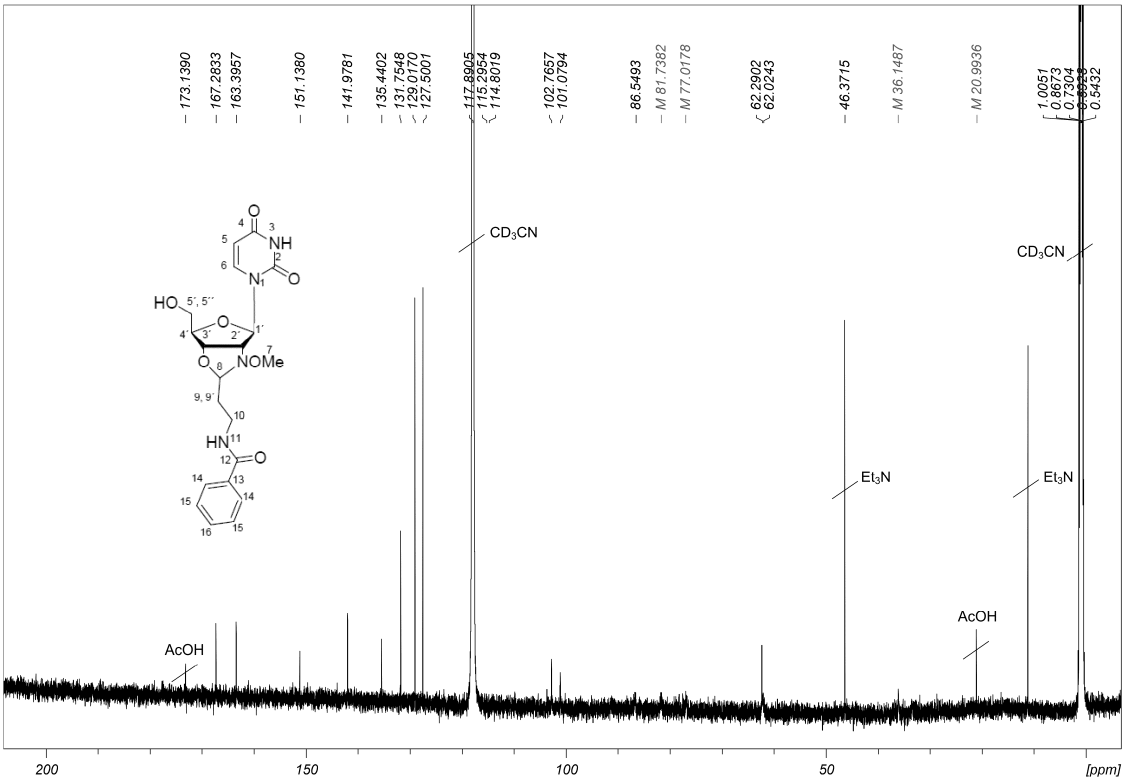

Figure S14. ^{13}C NMR (125 MHz, CD_3CN) spectrum of the *N*-Bz-β-Ala-H minor ligation product.

9. Determining hydrolysis rates of ASO-U^{NOMe} conjugates C1, C2, C3, and C4

Buffer solutions used in hydrolysis rate studies:

0.1 M AcOH, pH 4 at 37 °C (adjusted using NaOH), I = 0.1 L mol^{-1} (adjusted using NaCl)
0.1 M AcOH, pH 5 at 37 °C (adjusted using NaOH), I = 0.1 L mol^{-1} (adjusted using NaCl)
0.1 M MES, pH 6 at 37 °C (adjusted using NaOH), I = 0.1 L mol^{-1} (adjusted using NaCl)
0.1 M phosphate, pH 7.4 at 37 °C (adjusted using NaOH), I = 0.1 L mol^{-1} (adjusted using NaCl)

Conjugates **C1**, **C2**, **C3**, **C4** (10 μM) were incubated in aq. buffered solution (400 μL) at 37 ± 0.1 °C using a thermostated water bath. Samples were taken at adequate intervals and quenched by neutralizing with aqueous NaOH (0.4 M). Quenched samples were analyzed using RP HPLC (conditions in **Figure 3**). The amount of oligonucleotide released (by hydrolysis of the *N*-(methoxy)oxazolidine linker) at a specified time point was determined by comparing the relative peak areas of the released oligonucleotide to the conjugate. The half-life for the hydrolysis was determined according to the 1st order reaction rate law (cf. **Figure S15** and **Figure 2** for reaction profiles and **Figure 3** for selected RP HPLC profiles during hydrolysis reactions).

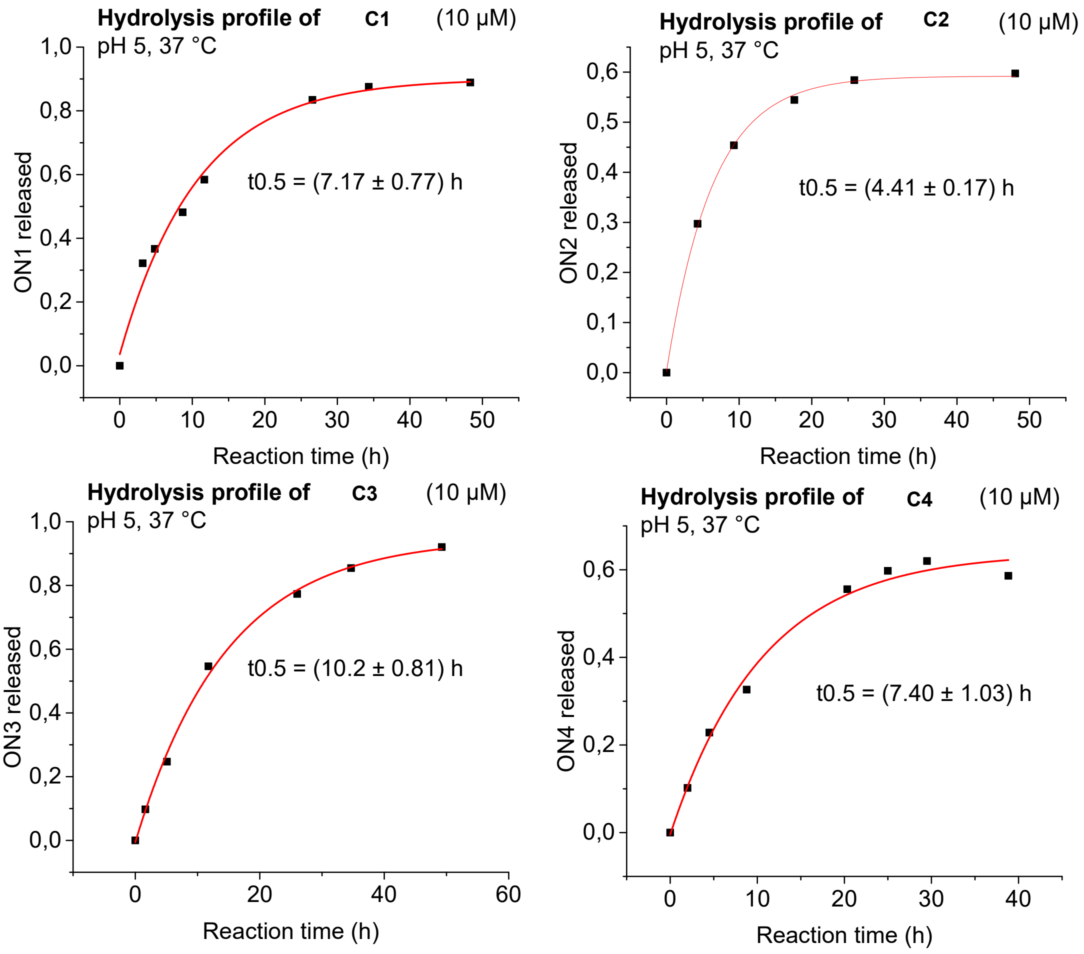

Figure S15. Hydrolysis reaction profiles of **U**^{NOMe}-conjugates **C1**, **C2**, **C3**, and **C4**.

10. NMR spectra of compounds 5, 6, and 7

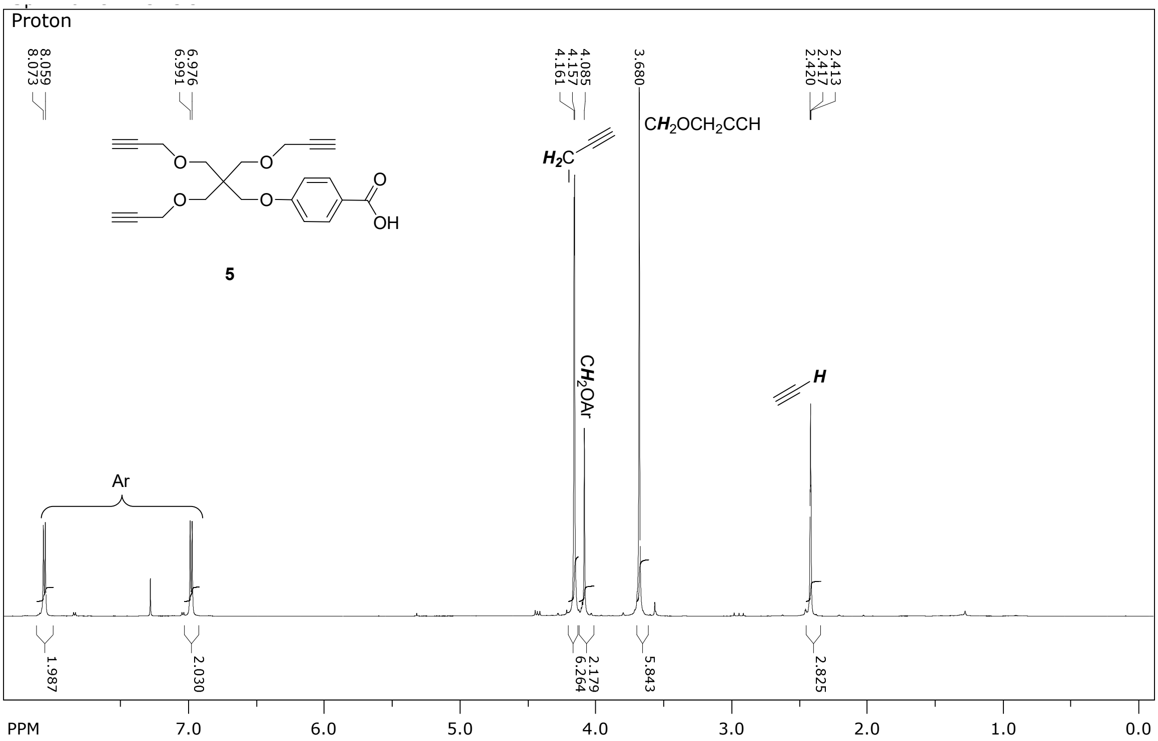

Figure S16. ^{1}H NMR (600 MHz, $CDCl_3$) spectrum of compound **5**.

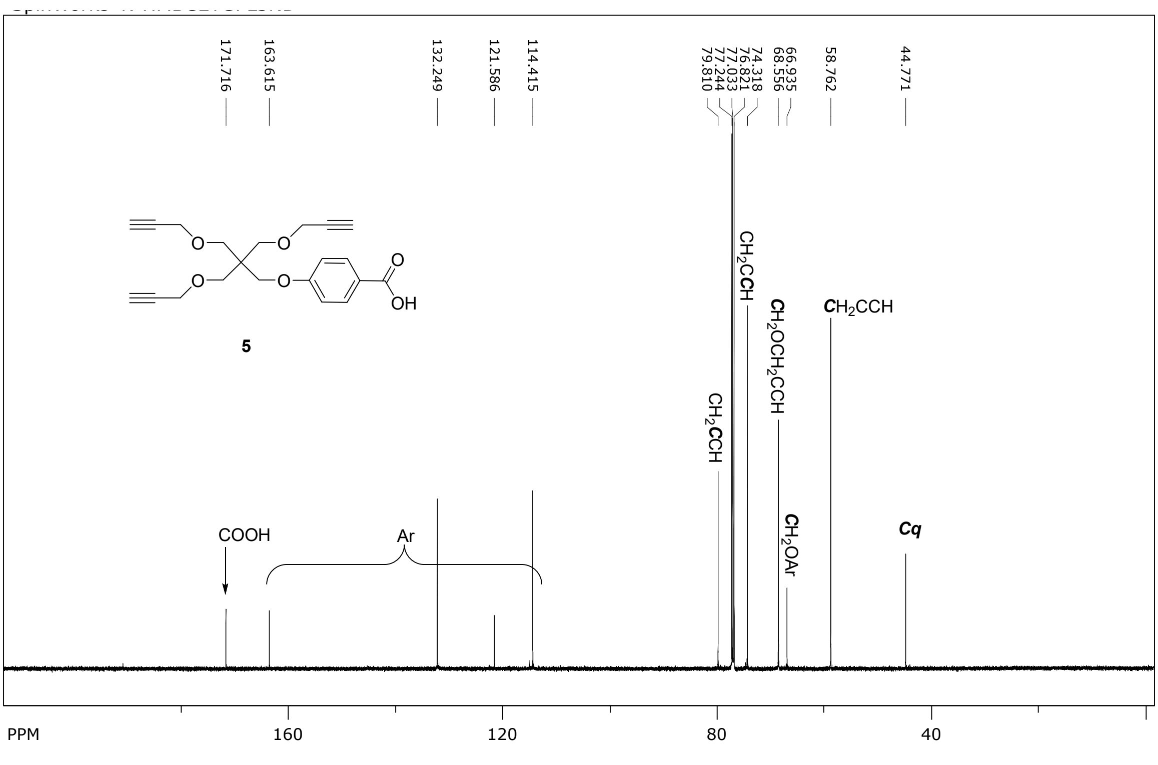

Figure S17. ^{13}C NMR (125MHz, $CDCl_3$) spectrum of compound **5**.

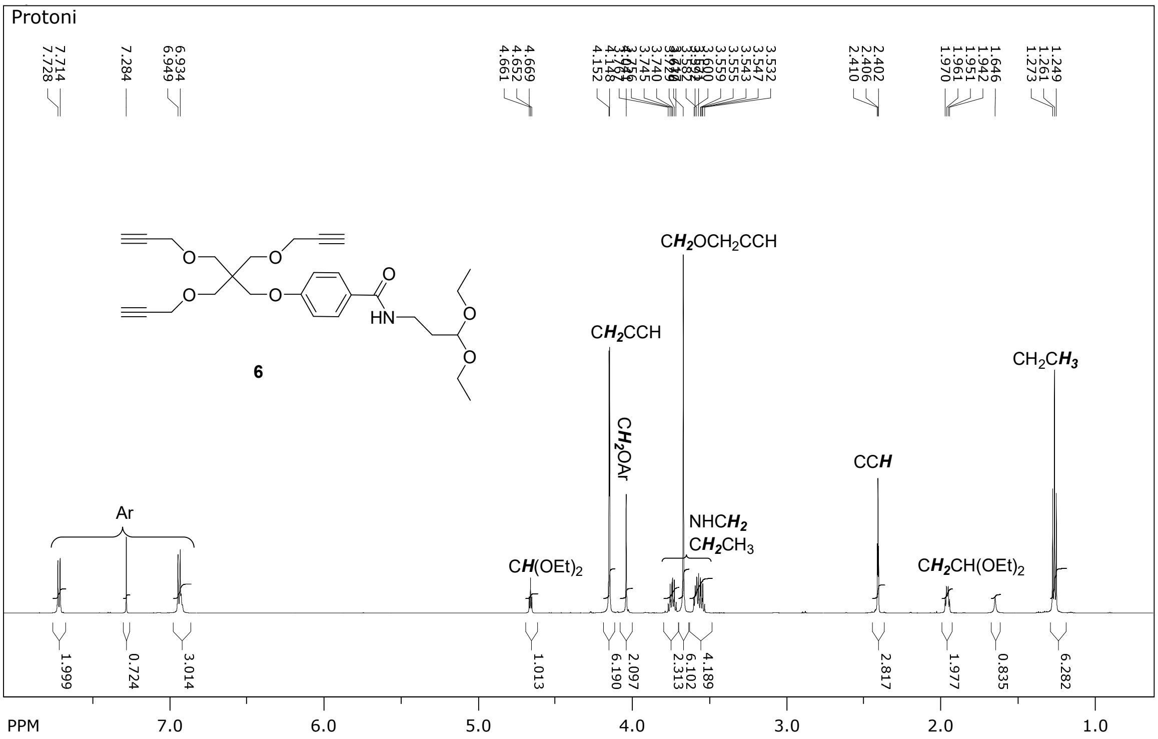

Figure S18. ^{1}H NMR (600 MHz, $CDCl_3$) spectrum of compound **6**.

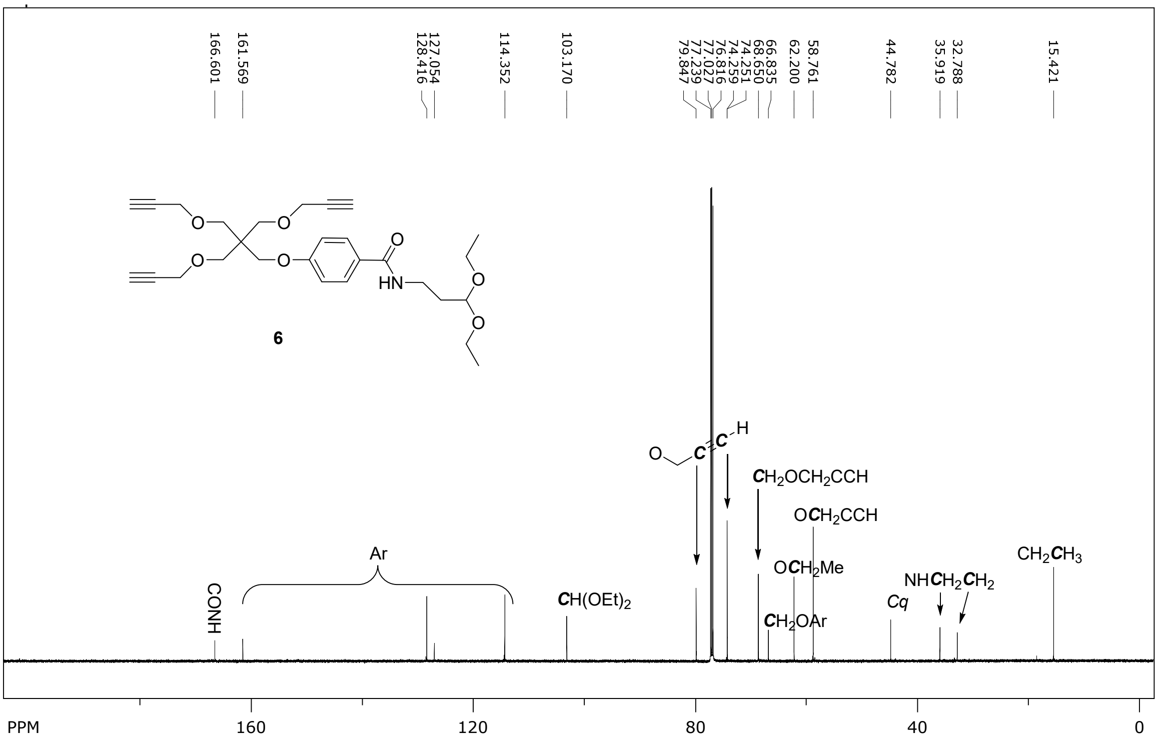

Figure S19. ^{13}C NMR (125MHz, $CDCl_3$) spectrum of compound **6**.

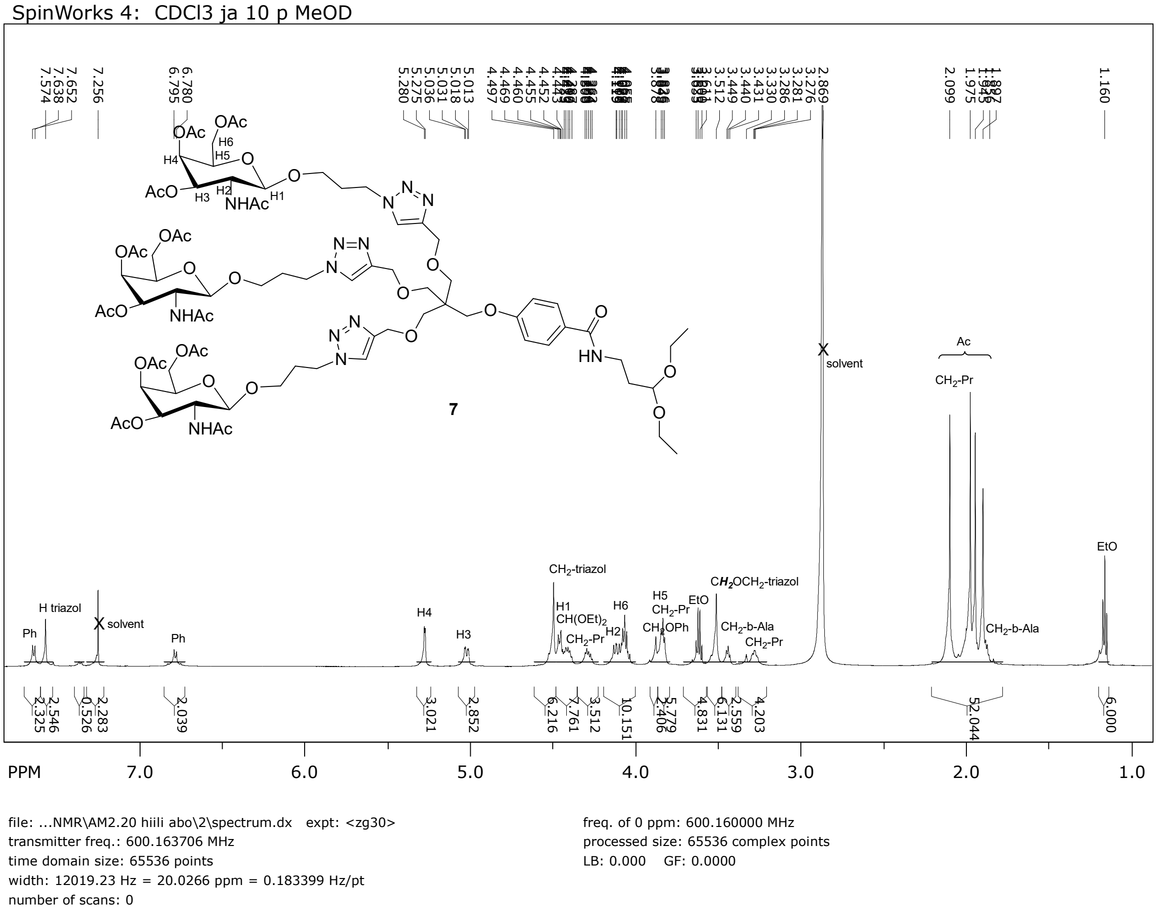

Figure S20. ^{1}H NMR (600MHz, MeOD-$CDCl_3$, 1:9, *v*/*v*) spectrum of compound **7**.

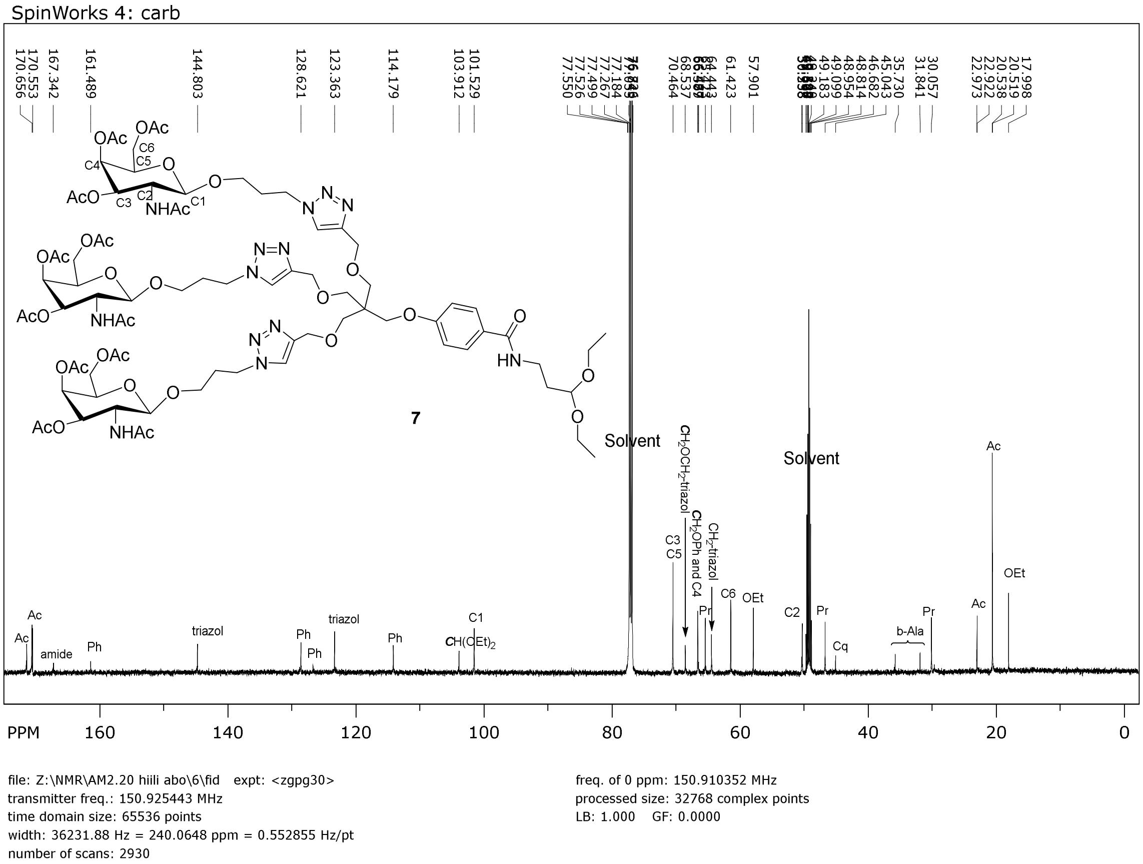

Figure S21. ^{13}C NMR (150MHz, MeOD-CDCl$_3$, 1:9, *v/v*) spectrum of compound **7**.

1. Häring, A. P.; Biallas, P.; Kirsch, S. F. An Unconventional Reaction of 2,2-Diazido Acylacetates with Amines. *Eur. J. Org. Chem.* **2017**, *2017*, 1526-1539.
2. Laitar, D. S.; Kramer, J. W.; Whiting, B. T.; Lobkovsky, E. B.; Coates, G. W. β-Amidoaldehydes via oxazoline hydroformylation. *Chem. Commun.* **2009**, 5704-5706.
3. Leyerer, K.; Koppermann, S.; Ducho, C. Solid Phase-Supported Synthesis of Muraymycin Analogues. *Eur. J. Org. Chem.* **2019**, *2019*, 7420-7431.
4. Aho, A.; Sulkanen, M.; Korhonen, H.; Virta, P. Conjugation of Oligonucleotides to Peptide Aldehydes via a pH-Responsive N-Methoxyoxazolidine Linker. *Org. Lett.* **2020**, *22*, 6714-6718.

(5) Determined by NMR quantification and UV-titration.
